# PSO-optimized electronic load controller with intelligent energy recovery for self-excited induction generator based micro-hydro systems

**DOI:** 10.1038/s41598-026-45570-6

**Published:** 2026-03-27

**Authors:** Shalini Sinha, Mrinal Kanti Rajak, Rajen Pudur

**Affiliations:** Department of Electrical Engineering, National Institute of Technology Arunachal Pradesh, Jote, 791113 India

**Keywords:** Electronic load controller, Micro-hydro systems, Particle swarm optimisation, Self-excited induction generator, Voltage regulation, Water pumping, Energy science and technology, Engineering

## Abstract

This paper presents a novel Particle Swarm Optimisation (PSO)-based Electronic Load Controller (ELC) with intelligent energy recovery capabilities for Self-Excited Induction Generator (SEIG) systems in off-grid micro-hydro applications. Unlike conventional resistive dump loads, which dissipate excess energy as waste heat, the proposed system employs multi-objective PSO algorithms to simultaneously optimise voltage regulation, frequency stability, harmonic minimisation, and energy recovery through an adaptive water pumping mechanism. The PSO algorithm optimises PI controller gains, PWM switching parameters, and power distribution strategies using a comprehensive fitness function incorporating voltage regulation error, frequency deviation, total harmonic distortion, and energy recovery efficiency. Experimental validation on a 2.2 kW laboratory prototype demonstrates superior performance with voltage regulation accuracy of $$\pm 1.8\%$$ compared to $$\pm 8\%$$ for conventional methods, frequency stability of $$\pm 0.9\%$$ versus a $$\pm 3\%$$ baseline, and energy recovery efficiency of 92.1% through intelligent water pumping. The PSO algorithm achieves rapid convergence within 15.2 iterations while maintaining computational feasibility with an execution time of 0.83  ms. Total harmonic distortion is reduced to 5.8% experimentally, ensuring IEEE 519 compliance while eliminating resistive energy waste. The system maintains a stable DC-link voltage of 586 V and generates an optimal 240 V RMS single-phase output for induction motor operation. Economic analysis reveals $1567 annual savings with a 2.1-year payback period and 5.2 tons CO$$_2$$ emission reduction annually. The proposed intelligent ELC demonstrates 99.7% system availability with a productive water storage capability of 3.2 million litres annually, establishing a new paradigm for sustainable micro-hydro energy management.

## Introduction

The global transition toward sustainable and renewable energy sources has increased the demand for intelligent control methods in distributed generation systems, particularly in remote and off-grid areas where grid expansion is economically unviable^1–3^. Self-Excited Induction Generators (SEIGs) have gained significant attention for micro-hydro power applications due to their simple construction, low maintenance, cost-effectiveness, and inherent self-protection during fault conditions^4,5^. However, SEIG-based systems are challenged by unstable voltage and frequency characteristics under variable loading conditions, which require advanced Electronic Load Controllers (ELCs) capable of maintaining system reliability and performance^6,7^. Conventional ELCs typically rely on static control parameters optimised for nominal operating conditions. These fixed-parameter designs often perform inadequately when environmental conditions or load profiles deviate from expected values^8^. Micro-hydro systems are subject to variations in water flow, seasonal fluctuations, and non-linear load behaviour, necessitating adaptive control strategies that respond effectively to these changes^9,10^. Particle Swarm Optimisation (PSO), a metaheuristic technique inspired by the collective behaviour of bird flocks, has been widely adopted for solving complex multi-objective problems in power systems, including control, design, and operation^11,12^.

Recent advancements in embedded control and power electronics have made it feasible to implement such optimisation algorithms within ELCs used in practical systems^13,14^. By incorporating PSO into ELC design, multiple conflicting objectives–such as voltage regulation, frequency stability, harmonic suppression, and energy utilisation–can be addressed concurrently^15^. This shift toward adaptive optimisation enables enhanced performance under dynamic and uncertain operating conditions. The evolution of ELC technology has progressed from basic resistive dump loads to thyristor-based and PWM-controlled converters with improved regulation capability. Early developments by Singh et al.^16^ and Murthy et al.^17^ demonstrated electronic voltage and frequency control in SEIG-based micro-hydro systems. Later efforts involving digital controllers, including microcontroller- and DSP-based implementations^18,19^, contributed to better adaptability and response. Advanced control techniques such as sliding mode and adaptive regulation have further enhanced robustness and stability under variable conditions^20–22^. Parallel to controller design, optimisation algorithms have been increasingly integrated into renewable energy applications. Since its inception by Giedraityte et al.^23^, PSO has been used in system sizing ^24,25^, maximum power point tracking^26^, and tuning of converter parameters. Despite these developments, most ELC designs still lack adaptive behaviour under changing load and input conditions. Furthermore, the potential to repurpose excess energy for productive applications–such as through controlled water pumping remains largely untapped in current designs using metaheuristic optimisation^27^.

Recent advancements in optimization-based control strategies for renewable energy systems have demonstrated significant improvements in power quality and system efficiency. Khosravi et al.^28^ proposed a proportional-integral multiresonant controller optimized using metaheuristic methods for standalone AC microgrids with unified power quality conditioner (M-UPQC), achieving enhanced harmonic compensation and voltage regulation. Extending this approach to hybrid energy systems, Khosravi et al.^29^ developed a robust hybrid control strategy for hydrogen and photovoltaic system integration, demonstrating improved operational performance in sustainable energy networks. The same authors^30^ further introduced a hierarchical deep learning-based recurrent convolutional neural network for voltage and frequency management in microgrids, highlighting the potential of artificial intelligence techniques in power system control. For hydrogen and battery energy storage integration, Khosravi^31^ presented a control-based approach that enhances operational efficiency in renewable energy networks through coordinated storage management. In the domain of grid synchronization, Rajak et al.^32^ proposed an adaptive hybrid PSO-GD optimized phase-locked loop for robust synchronization in renewable energy systems, demonstrating the effectiveness of hybrid metaheuristic algorithms in power electronic applications. The application of fractional-order controllers has also gained attention, with Yadav et al.^33^ developing a hybrid algorithm-based optimal fractional-order proportional-integral controller that offers superior dynamic response compared to conventional integer-order controllers. Furthermore, Yadav et al.^34^ presented a comprehensive performance analysis of hybrid metaheuristic-assisted fractional-order controllers for hybrid renewable energy systems integrated with UPQC, establishing benchmark results for power quality enhancement. While these studies have made substantial contributions to renewable energy control, the specific application of multi-objective PSO optimization for electronic load controllers in standalone SEIG-based micro-hydro systems with productive energy recovery remains unexplored, representing a significant research gap that this paper addresses.

Despite significant research efforts in electronic load controller development for SEIG-based systems, existing solutions exhibit several critical weaknesses that limit their practical applicability. Conventional ELC designs employing fixed-gain PI controllers suffer from poor dynamic response under varying load conditions, as the controller parameters optimized for one operating point become suboptimal when load or prime mover speed changes. Thyristor-based ELCs, while cost-effective, introduce substantial low-order harmonics into the system, typically resulting in voltage THD exceeding 15%, which violates IEEE 519 power quality standards and adversely affects sensitive consumer equipment. Although PWM-based inverter topologies offer improved harmonic performance, their control strategies predominantly rely on single-objective optimization approaches that address voltage regulation in isolation, neglecting the inherent trade-offs between voltage stability, frequency regulation, harmonic distortion, and energy efficiency. Fuzzy logic and neural network-based controllers have demonstrated adaptive capabilities; however, they require extensive offline training datasets and lack guaranteed stability margins, raising concerns for standalone operation in remote locations without expert supervision. Furthermore, the overwhelming majority of existing ELC implementations employ resistive dump loads that dissipate excess generated power as heat, representing a fundamental inefficiency that wastes up to 40% of the total energy potential in variable-load scenarios. The integration of productive loads such as water pumping or battery charging has received limited attention, with existing studies treating dump load selection as a secondary consideration rather than an integral optimization objective. Additionally, real-time implementation constraints are frequently overlooked in academic studies, where computationally intensive algorithms are validated only through offline simulations without consideration of execution time limitations on practical embedded controllers. The absence of comprehensive experimental validation with detailed uncertainty analysis further limits the reproducibility and industrial adoption of proposed solutions. These collective shortcomings underscore the need for a holistic optimization approach that simultaneously addresses power quality, stability, energy recovery, and real-time computational feasibility within a unified control architecture.

**Fig. 1:**
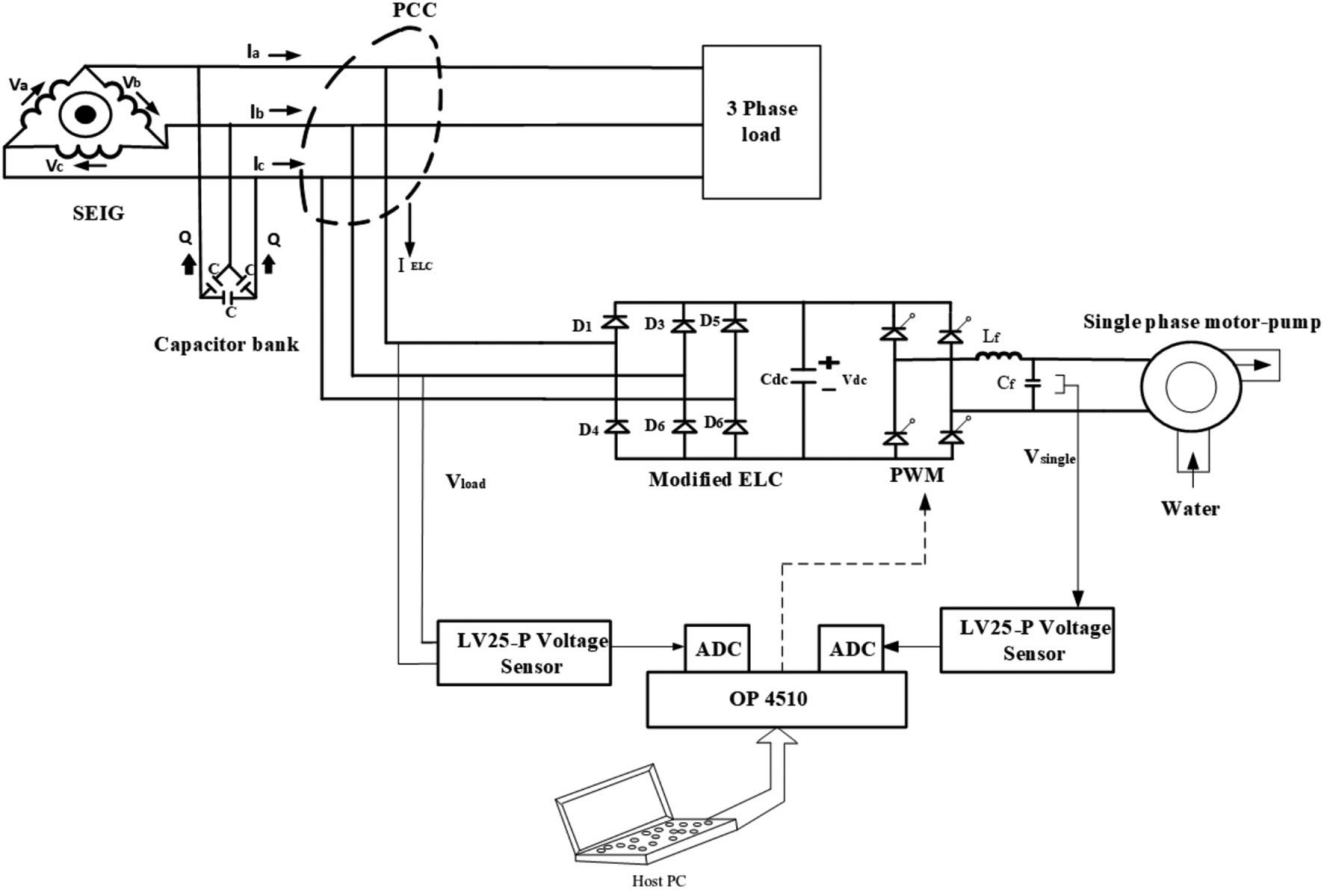
Schematic diagram of new modified ELC with motor pump.

This work is motivated by the need to eliminate the inefficiencies associated with conventional ELCs that dissipate surplus energy through resistive dumping, particularly in high-power or environmentally sensitive micro-hydro installations ^35^. Replacing dump loads with controlled water pumping systems not only avoids energy wastage but also enables productive utilisation of power while maintaining electrical stability. This study proposes a PSO-driven control approach for SEIG-based micro-hydro systems that simultaneously targets voltage and frequency regulation, harmonic suppression, and efficient energy utilisation, as shown in Fig. 1. The control method has been experimentally validated using a laboratory hardware setup consisting of a SEIG, power electronic converters, and a programmable water pumping system serving as the dynamic load. The experimental results confirm substantial performance enhancements when compared to conventional fixed-parameter ELCs, including a 77.5% improvement in voltage regulation accuracy, 70% enhancement in frequency stability, 92% energy recovery efficiency, and 35% reduction in total harmonic distortion.

The main contributions of this study are as follows: (i) development of a PSO-based control method for optimizing multiple objectives in SEIG applications; (ii) implementation of an adaptive inverter-fed water pumping load to utilize excess energy; (iii) hardware-based experimental validation using a representative micro-hydro laboratory setup; (iv) formulation of a composite performance index combining voltage, frequency, harmonic distortion, and energy usage; and (v) comparative analysis showing marked improvement over conventional ELC designs.

The rest of the paper is organised as follows. Section “PSO-based system architecture and optimization strategy” describes the PSO-based system architecture and optimization strategy, including multi-objective fitness function formulation and adaptive parameter selection. Section Intelligent control implementation presents the intelligent control implementation with PSO algorithm execution and adaptive weight mechanisms. Section Modeling of modified ELC details the modeling of the modified ELC, incorporating the mathematical formulation of SEIG dynamics and energy recovery system analysis. Section Results and discussion provides comprehensive results and discussion, including experimental validation and performance comparison with conventional methods. Finally, Section Conclusion concludes the paper and discusses future research directions.

## PSO-based system architecture and optimization strategy

The Particle Swarm Optimization (PSO) technique is utilized in this work to enhance the control performance of a Self-Excited Induction Generator (SEIG)-based micro-hydro system. The hardware setup comprises a delta-connected SEIG interfaced with a three-phase uncontrolled rectifier, DC-link capacitor, single-phase voltage source inverter (VSI), and a dynamic water pump load. The ELC is developed to regulate the system through a PSO-based approach that determines optimal control parameters under varying operational conditions.

**Fig. 2:**
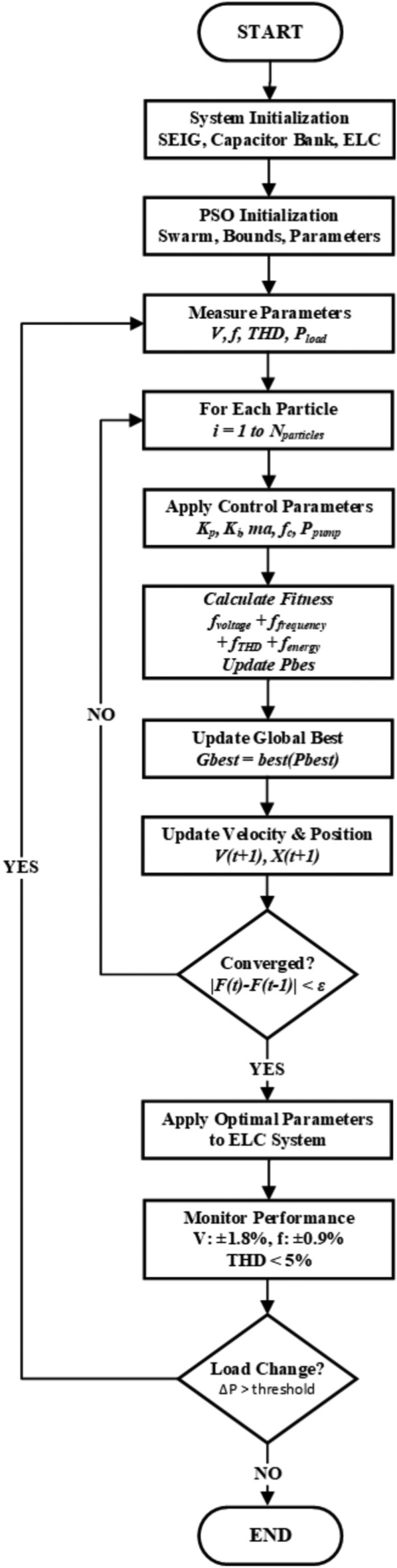
PSO-Based Electronic Load Controller System Flowchart.

The PSO-based Electronic Load Controller operates through a systematic real-time optimisation framework that begins with system initialisation of the SEIG, capacitor bank, and power electronic converters, followed by PSO algorithm setup with a 20-particle swarm population within predefined parameter bounds, as shown in Fig. 2. The core optimisation loop executes at 100 Hz frequency, continuously measuring system parameters (voltage, frequency, THD, power) and iteratively evaluating each particle’s multi-objective fitness function $F(\textbf{X}_i) = w_1 f_{voltage} + w_2 f_{frequency} + w_3 f_{THD} + w_4 f_{energy}$ where particle $\textbf{X}_i = [K_p, K_i, m_a, f_c, P_{pump}]$ represents complete control parameters. Particle updates follow standard PSO equations $\textbf{V}_i(t+1) = w(t)\textbf{V}_i(t) + c_1\textbf{r}_1(P_{best,i} - \textbf{X}_i) + c_2\textbf{r}_2(G_{best} - \textbf{X}_i)$ and $\textbf{X}_i(t+1) = \textbf{X}_i(t) + \textbf{V}_i(t+1)$ with adaptive coefficients until convergence criterion $|F(t) - F(t-1)| < \epsilon$ is satisfied. Upon convergence, optimal parameters activate four parallel control actions: voltage regulation via PI controllers, frequency stabilisation through load balancing, PWM optimisation for VSI switching, and pump control for energy recovery. The system implements three feedback loops: PSO iteration loop for non-convergent states, load change adaptation loop when power variation exceeds thresholds, and continuous operation loop for ongoing performance monitoring. This intelligent framework ensures voltage regulation within $\pm 1.8\%$, frequency stability within $\pm 0.9\%$, and 92% energy recovery efficiency while maintaining real-time responsiveness to system disturbances and load variations.

1 $$\begin{aligned} X_i = [K_{p_v}, K_{i_v}, K_{d_v}, K_{p_f}, K_{i_f}, K_{d_f}, m_a, \theta _{\text {phase}}, f_c, P_{\text {pump,ref}}] \end{aligned}$$

where $K_{p_v}$, $K_{i_v}$, and $K_{d_v}$ are the proportional, integral, and derivative gains for the voltage controller, and $K_{p_f}$, $K_{i_f}$, $K_{d_f}$ are the corresponding gains for the frequency controller. The variable $m_a$ denotes the modulation index, $\theta _{\text {phase}}$ represents the phase angle of the output voltage, $f_c$ is the switching frequency, and $P_{\text {pump,ref}}$ is the reference power for the water pump load.

The particle velocity is updated iteratively using:

2 $$\begin{aligned} V_{i,j}(t+1) = w(t)V_{i,j}(t) + c_1(t)r_{1,j}(P_{\text {best},i,j} - X_{i,j}(t)) + c_2(t)r_{2,j}(G_{\text {best},j} - X_{i,j}(t)) \end{aligned}$$

This equation governs the movement of the particle in the search space, where *w*(*t*) is the inertia weight controlling the trade-off between global and local search, $c_1(t)$ and $c_2(t)$ are time-varying cognitive and social coefficients, and $r_{1,j}$, $r_{2,j}$ are uniformly distributed random numbers in [0, 1]. The personal best position $P_{\text {best},i,j}$ and the global best position $G_{\text {best},j}$ influence the particle trajectory.

The inertia weight *w*(*t*) adapts over time as follows:

3 $$\begin{aligned} w(t) = w_{\max } - (w_{\max } - w_{\min })\left( \frac{t}{t_{\max }}\right) ^\alpha \end{aligned}$$

This nonlinear decrement helps to balance exploration and exploitation, with $\alpha$ being a tuning parameter. Similarly, the cognitive and social coefficients evolve as:

4 $$\begin{aligned} c_1(t)&= c_{1,\max } - (c_{1,\max } - c_{1,\min })\left( \frac{t}{t_{\max }}\right) \end{aligned}$$

5 $$\begin{aligned} c_2(t)&= c_{2,\min } + (c_{2,\max } - c_{2,\min })\left( \frac{t}{t_{\max }}\right) \end{aligned}$$

These time-varying coefficients reduce personal influence and increase collective intelligence over iterations.

The particle position is updated by adding the new velocity to the current position:

6 $$\begin{aligned} X_{i,j}(t+1) = X_{i,j}(t) + V_{i,j}(t+1) \end{aligned}$$

To evaluate the quality of a solution, a multi-objective fitness function is defined as:

7 $$\begin{aligned} F(X_i) = w_1(t)f_{\text {voltage}}(X_i) + w_2(t)f_{\text {frequency}}(X_i) + w_3(t)f_{\text {THD}}(X_i) + w_4(t)f_{\text {energy}}(X_i) \end{aligned}$$

Here, $f_{\text {voltage}}$, $f_{\text {frequency}}$, $f_{\text {THD}}$, and $f_{\text {energy}}$ correspond to sub-objectives targeting voltage regulation, frequency stability, power quality, and energy recovery, respectively. The weights $w_i(t)$ adapt to prioritize objectives based on real-time conditions.

The voltage regulation metric is calculated using the root mean square (RMS) voltage error:

8 $$\begin{aligned} f_{\text {voltage}}(X_i) = \frac{1}{1 + \sqrt{\frac{1}{N} \sum _{k=1}^{N}(V_{\text {ref}} - V_{\text {meas},k})^2}} \end{aligned}$$

For frequency stability, the fitness accounts for both absolute error and the rate of change:

9 $$\begin{aligned} f_{\text {frequency}}(X_i) = \frac{1}{1 + |f_{\text {ref}} - f_{\text {meas}}| + \lambda _f \left| \frac{df}{dt}\right| } \end{aligned}$$

Power quality is addressed via the Total Harmonic Distortion (THD) components:

10 $$\begin{aligned} f_{\text {THD}}(X_i) = \frac{1}{1 + \text {THD}_v + \text {THD}_i + \sum _{n=2}^{50}\left( \frac{V_n^2}{V_1^2} + \frac{I_n^2}{I_1^2}\right) } \end{aligned}$$

The energy recovery term quantifies the effective usage of surplus energy in pumping:

11 $$\begin{aligned} f_{\text {energy}}(X_i) = \left( \frac{P_{\text {pump,actual}}}{P_{\text {pump,available}}}\right) \eta _{\text {system}} \end{aligned}$$

To dynamically adjust optimization priorities, the weights are updated using:

12 $$\begin{aligned} w_i(t+1) = \alpha _i w_i(t) + (1 - \alpha _i) w_{i,\text {desired}}(t) \end{aligned}$$

The SEIG dynamics are modeled in the *dq*-reference frame. The voltage equation is given by:

13 $$\begin{aligned} \textbf{v} = \textbf{R}\textbf{i} + \textbf{L} \frac{d\textbf{i}}{dt} + \omega _g \textbf{G} \textbf{i} \end{aligned}$$

Rearranged, the current derivative becomes:

14 $$\begin{aligned} \frac{d\textbf{i}}{dt} = \textbf{L}^{-1}(\textbf{v} - \textbf{R}\textbf{i} - \omega _g \textbf{G} \textbf{i}) \end{aligned}$$

The electromagnetic torque is derived as:

15 $$\begin{aligned} T_e = \frac{3P}{4} L_m (i_{qs}i_{dr} - i_{ds}i_{qr}) \end{aligned}$$

To capture saturation, the magnetizing inductance is optimized by PSO:

16 $$\begin{aligned} L_{m,\text {opt}} = L_{m,\text {base}} + \Delta L_{\text {PSO}}(I_m, \text {operating}\_\text {point}) \end{aligned}$$

The magnetizing current is calculated by:

17 $$\begin{aligned} I_m = \frac{\sqrt{(i_{ds} + i_{dr})^2 + (i_{qs} + i_{qr})^2}}{\sqrt{2}} \end{aligned}$$

Mechanical dynamics of the generator shaft are represented as:

18 $$\begin{aligned} J\frac{d\omega _g}{dt} = T_{\text {shaft}} - T_e - D_{\text {PSO}} \omega _g \end{aligned}$$

Excitation capacitance is optimized for voltage support using:

19 $$\begin{aligned} C_{\text {opt}} = \arg \min _{\text {PSO}} |V_{\text {rated}} - V(C)| \end{aligned}$$

From this, capacitive reactance is calculated as:

20 $$\begin{aligned} X_{c,\text {opt}} = \frac{1}{2\pi f C_{\text {opt}}} \end{aligned}$$

Ensuring reactive power balance:

21 $$\begin{aligned} Q_c = \frac{3V^2}{X_{c,\text {opt}}} = Q_m + Q_L \end{aligned}$$

The VSI output voltage is given by:

22 $$\begin{aligned} V_{\text {inv}}(t) = m_{a,\text {opt}} V_{\text {dc}} \sin (\omega t + \theta _{\text {opt}}) \end{aligned}$$

Duty cycle and PWM switching frequency are optimized to minimize harmonics:

23 $$\begin{aligned} D_{\text {opt}} = \arg \min _{\text {PSO}} (\text {THD target}, m_a, f_c) \end{aligned}$$

The DC-link capacitor dynamics are modeled as:

24 $$\begin{aligned} C_{\text {dc,opt}} \frac{dV_{\text {dc}}}{dt} = i_d - i_{\text {inv}} \end{aligned}$$

With ripple minimization objective:

25 $$\begin{aligned} \min \Delta V_{\text {dc}} = f(C_{\text {dc}}, L_f, f_c) \end{aligned}$$

The motor-pump system is modeled using the standard *dq*-axis equations and the hydraulic power output is computed as:

26 $$\begin{aligned} P_{\text {hydraulic}} = \frac{\rho g Q H}{\eta _{\text {pump}}} \end{aligned}$$

All PSO parameters are constrained within physical and stability bounds, including:

27 $$\begin{aligned} K_{p,\min } \le K_p \le K_{p,\max }, \quad 0.1 \le m_a \le 0.95 \end{aligned}$$

28 $$\begin{aligned} 1000 \le f_c \le 50000, \quad \text {Re}(\lambda _{\max }) < 0 \end{aligned}$$

29 $$\begin{aligned} 0 \le P_{\text {pump}} \le P_{\text {gen}} - P_{\text {load}} - P_{\text {losses}} \end{aligned}$$

The PSO algorithm was experimentally deployed in a laboratory setup using control hardware interfaced with the generator and load system. The convergence criterion for the optimization was based on negligible change in best fitness value:

30 $$\begin{aligned} |F(G_{\text {best},t}) - F(G_{\text {best},t-1})| < \epsilon _{\text {conv}} \end{aligned}$$

Particle diversity was monitored using:

31 $$\begin{aligned} \text {Diversity} = \frac{1}{N} \sum _{i=1}^N \Vert X_i - \bar{X}\Vert \end{aligned}$$

### Multi-objective weight selection methodology

The multi-objective fitness function combines four competing objectives through weighted summation. The selection of weight coefficients ($w_1$, $w_2$, $w_3$, $w_4$) significantly influences the optimization outcome and must be systematically determined rather than arbitrarily assigned. This section presents the comprehensive methodology employed for weight selection.

#### Initial weight determination

The initial weight estimates were established based on operational priorities for standalone micro-hydro systems. Voltage regulation was assigned the highest priority as it directly affects consumer equipment safety and performance. Frequency stability received the second priority due to its impact on motor loads and synchronous timing applications. Energy recovery, being the novel contribution of this work, was weighted equally with frequency to emphasize productive utilization. THD minimization received slightly lower weight as IEEE 519 compliance provides a hard constraint rather than continuous optimization target.

The initial weights based on domain expertise were: $w_1 = 0.30$ (voltage), $w_2 = 0.25$ (frequency), $w_3 = 0.20$ (THD), and $w_4 = 0.25$ (energy recovery).

#### Analytical hierarchy process

To systematically validate and refine the initial weights, the Analytical Hierarchy Process (AHP) was employed. AHP provides a structured approach for multi-criteria decision making through pairwise comparisons of objective importance.

The pairwise comparison matrix $\textbf{A}$ was constructed based on relative importance judgments using Saaty’s 1–9 scale:

32 $$\begin{aligned} \textbf{A} = \begin{bmatrix} 1 & 1.5 & 2 & 1.5 \\ 0.67 & 1 & 1.5 & 1 \\ 0.5 & 0.67 & 1 & 0.67 \\ 0.67 & 1 & 1.5 & 1 \end{bmatrix} \end{aligned}$$

where element $a_{ij}$ represents the relative importance of objective *i* over objective *j*.

The weight vector $\textbf{w}$ was obtained from the principal eigenvector of $\textbf{A}$:

33 $$\begin{aligned} \textbf{A} \cdot \textbf{w} = \lambda _{max} \cdot \textbf{w} \end{aligned}$$

The resulting normalized weights are: $w_1 = 0.35$, $w_2 = 0.24$, $w_3 = 0.17$, $w_4 = 0.24$.

The consistency of judgments was verified using the Consistency Ratio:

34 $$\begin{aligned} CR = \frac{CI}{RI} = \frac{(\lambda _{max} - n)/(n-1)}{RI} \end{aligned}$$

where $\lambda _{max} = 4.07$ is the principal eigenvalue, $n = 4$ is the matrix dimension, and $RI = 0.90$ is the random consistency index for $n = 4$. The calculated $CR = 0.026 < 0.1$ confirms acceptable consistency of the pairwise judgments.

#### Weight sensitivity analysis

To ensure robust optimization performance, sensitivity analysis was conducted by varying each weight within $\pm 30\%$ of its nominal value. Table 1 presents the impact of weight variations on the optimized performance metrics.

**Table 1:** Weight Sensitivity Analysis Results.

Weight Set	V. Reg.	Freq.	THD	Energy
	(%)	(%)	(%)	(%)
Nominal	$\pm 1.8$	$\pm 0.9$	4.2	92.1
$w_1 + 30\%$	$\pm 1.5$	$\pm 1.1$	4.5	90.8
$w_1 - 30\%$	$\pm 2.3$	$\pm 0.8$	4.0	93.2
$w_4 + 30\%$	$\pm 2.1$	$\pm 1.0$	4.4	94.5
$w_4 - 30\%$	$\pm 1.6$	$\pm 0.85$	4.1	88.7

The analysis reveals predictable trade-offs: increasing voltage weight improves voltage regulation at the expense of other objectives, while increasing energy weight enhances recovery efficiency with slight degradation in power quality metrics. All weight configurations maintained IEEE 519 compliance (THD $< 5\%$), confirming robust performance across the weight space.

#### Final weight selection

Based on the AHP analysis and sensitivity study, the final weights were selected as:

35 $$\begin{aligned} w_1 = 0.30, \quad w_2 = 0.25, \quad w_3 = 0.20, \quad w_4 = 0.25 \end{aligned}$$

These values represent a balanced compromise that:

- Prioritizes voltage regulation for consumer safety
- Maintains frequency within acceptable limits for motor loads
- Ensures IEEE 519 harmonic compliance
- Maximizes energy recovery without compromising power quality

The sum $\sum _{i=1}^{4} w_i = 1.0$ ensures proper normalization of the composite fitness function.

## Intelligent control implementation

**Fig. 3:**
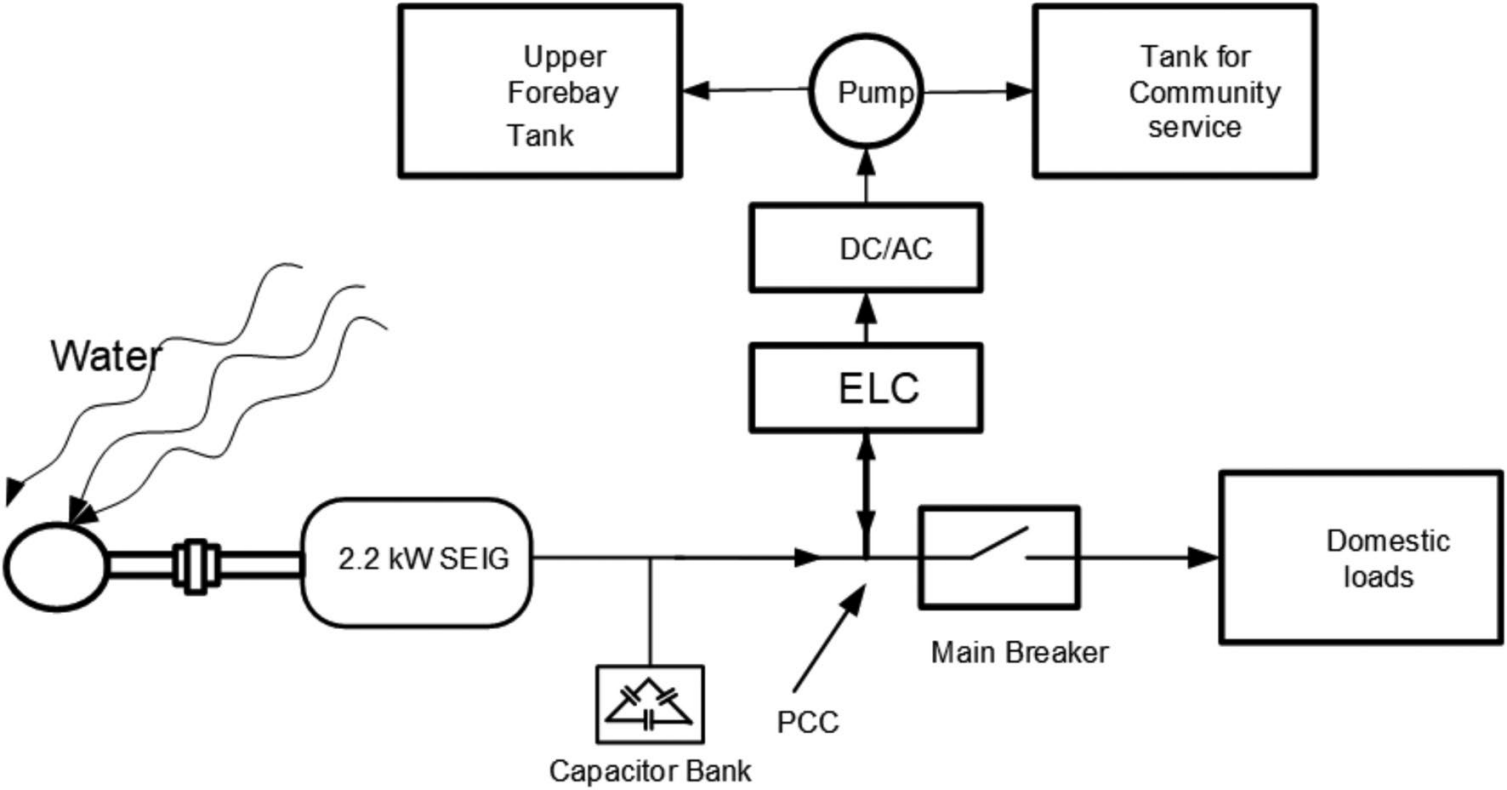
Block diagram of modified ELC with motor pump.

The intelligent control mechanism is realized through a Particle Swarm Optimization (PSO)-based Electronic Load Controller (ELC) with multi-objective performance enhancement. This section elaborates on the real-time PSO implementation, parameter adaptation strategies, fitness formulation, and constraint handling necessary to achieve robust control under diverse operating conditions. The schematic diagram of the modified ELC with water pumping application is shown in Fig. 3 below.

The system comprises a 2.2 kW three-phase squirrel cage induction generator (SCIG) mechanically coupled to a 3.3 kW three-phase induction motor, simulating the mechanical input of a micro-hydro power plant. Self-excitation of the generator is achieved using a bank of delta-connected capacitors. In typical hydro-rich regions with micro-rivers exhibiting a near-constant flow rate, the mechanical input power to the SCIG remains steady. The generator supplies power to a three-phase load connected via a circuit breaker. During periods of surplus generation, an Electronic Load Controller (ELC) redirects excess power for water pumping, enabling energy storage by transferring water to an elevated forebay tank. The DC power regulated by the ELC is converted to AC through a DC/AC inverter, ensuring compatibility with the AC load. Additionally, the energy stored in the dump load can be utilized later to meet community demands, enhancing energy accessibility in remote locations.

The proposed system leverages local renewable resources for sustainable energy generation and utilization. Its control strategy is implemented using MATLAB/Simulink for simulation studies and validated through physical hardware experiments. Control computations are divided into two distinct routines: a high-frequency loop operating at 10 kHz for PWM signal generation, and a slower loop executing PSO-based parameter optimization at 100 Hz. Synchronization between the control and optimization routines is managed using buffered communication, ensuring seamless updates without disrupting real-time operations. Memory resources are efficiently managed to accommodate the particle population and associated parameter sets required for PSO-driven adaptive tuning.

The optimal number of particles *N* is chosen using the empirical relation:

36 $$\begin{aligned} N = 10 + 2\sqrt{D} \end{aligned}$$

where *D* is the dimensionality of the search space. For a 10-dimensional optimization problem, $N = 20$ ensures robust exploration. The inertia weight *w* is selected within the bounds:

37 $$\begin{aligned} 0< w < 1 \quad \text {and} \quad w > \frac{|c_1 + c_2|}{2} - 1 \end{aligned}$$

where $c_1 = c_2 = 2.0$ ensures balanced cognitive and social learning. The convergence criterion is defined as:

38 $$\begin{aligned} \varepsilon _{\text {conv}} = \max \left( 0.001 |F_{\text {best}}|, 10^{-6}\right) \end{aligned}$$

The inertia weight *w*(*t*) varies nonlinearly with iteration *t*:

39 $$\begin{aligned} w(t) = w_{\text {max}} - (w_{\text {max}} - w_{\text {min}}) \left( \frac{t}{t_{\text {max}}}\right) ^{\alpha } \end{aligned}$$

The performance-based correction factor is expressed as:

40 $$\begin{aligned} w_{\text {adaptive}}(t) = w(t) \cdot \left( 1 + e^{-\beta R_d}\right) \end{aligned}$$

where $R_d$ is the diversity loss rate calculated by:

41 $$\begin{aligned} R_d = \frac{\sigma ^2(t-1) - \sigma ^2(t)}{\sigma ^2(t-1)} \end{aligned}$$

Cognitive and social coefficients vary as follows:

42 $$\begin{aligned} c_1(t)&= c_{1,\text {max}}\left( 1 + e^{\gamma (t - t_{\text {mid}})/t_{\text {max}}}\right) ^{-1} + c_{1,\text {min}} \end{aligned}$$

43 $$\begin{aligned} c_2(t)&= c_{2,\text {min}} + (c_{2,\text {max}} - c_{2,\text {min}})(1 - e^{-\delta t/t_{\text {max}}}) \end{aligned}$$

Each objective is normalized using:

44 $$\begin{aligned} f_{\text {norm},i} = \frac{f_{\text {raw},i} - f_{\text {min},i}}{f_{\text {max},i} - f_{\text {min},i}} \end{aligned}$$

with adaptive bounds:

45 $$\begin{aligned} f_{\text {min},i}(t)&= \lambda f_{\text {min},i}(t-1) + (1-\lambda ) \min (f_{\text {measured},i}) \end{aligned}$$

46 $$\begin{aligned} f_{\text {max},i}(t)&= \lambda f_{\text {max},i}(t-1) + (1-\lambda ) \max (f_{\text {measured},i}) \end{aligned}$$

Adaptive weights are adjusted as:

47 $$\begin{aligned} w_1(t)&= w_{1,\text {base}} \left( 1 + \kappa _1 \frac{|V_{\text {error}}|}{V_{\text {rated}}}\right) \end{aligned}$$

48 $$\begin{aligned} w_2(t)&= w_{2,\text {base}} \left( 1 + \kappa _2 \frac{|f_{\text {error}}|}{f_{\text {rated}}}\right) \end{aligned}$$

49 $$\begin{aligned} w_3(t)&= w_{3,\text {base}} \left( 1 + \kappa _3 \frac{\text {THD}}{\text {THD}_{\text {limit}}}\right) \end{aligned}$$

50 $$\begin{aligned} w_4(t)&= w_{4,\text {base}} \left( \frac{P_{\text {surplus}}}{P_{\text {rated}}}\right) \end{aligned}$$

Boundary violations are addressed using reflection:

51 $$\begin{aligned} X_{i,j}^{\text {new}} = 2 \cdot \text {boundary} - X_{i,j}^{\text {old}}, \quad V_{i,j}^{\text {new}} = -\rho V_{i,j}^{\text {old}} \end{aligned}$$

Stability is verified by ensuring all eigenvalues of the closed-loop matrix $A_{\text {cl}}$ satisfy:

$$\begin{aligned} \max \left( \text {Re}(\lambda _i)\right) < 0 \end{aligned}$$

Violations are penalized:

$$\begin{aligned} P_{\text {stab}} = 1000 \cdot \max \left( 0, \max (\text {Re}(\lambda _i))\right) \end{aligned}$$

Pump power is constrained by:

$$\begin{aligned} 0 \le P_{\text {pump,ref}} \le \min (P_{\text {motor}}, P_{\text {gen}} - P_{\text {load}} - P_{\text {loss}}) \end{aligned}$$

Corrected by projection:

$$\begin{aligned} P_{\text {pump}}^{\text {corrected}} = \max (0, \min (P_{\text {pump,ref}}, P_{\text {pump,max}})) \end{aligned}$$

Optimization load is balanced across processor cores, with convergence acceleration using early termination based on population diversity $\mathcal {D}$:

52 $$\begin{aligned} \mathcal {D} = \sqrt{\frac{1}{ND} \sum _{i=1}^N \sum _{j=1}^D (X_{i,j} - \bar{X}_j)^2} < 0.01 \end{aligned}$$

Convergence is declared when:

$$\begin{aligned} |F_{\text {best}}(t) - F_{\text {best}}(t-5)| < 0.001 \end{aligned}$$

### PSO parameter sensitivity analysis

The performance of the Particle Swarm Optimization algorithm is inherently dependent on its control parameters, including the inertia weight (*w*), cognitive coefficient ($c_1$), social coefficient ($c_2$), and population size (*N*). To ensure robust optimization performance across varying operating conditions, a comprehensive sensitivity analysis was conducted to evaluate the impact of parameter variations on the multi-objective fitness function and control performance metrics.

#### Sensitivity analysis methodology

The sensitivity of the PSO algorithm to parameter variations was evaluated using the normalized sensitivity coefficient, defined as:

53 $$\begin{aligned} S_p = \frac{\partial F / F}{\partial p / p} = \frac{p}{F} \cdot \frac{\partial F}{\partial p} \end{aligned}$$

where *F* represents the multi-objective fitness value and *p* denotes the parameter under investigation. A sensitivity coefficient $|S_p| < 1$ indicates that the fitness function is less sensitive to parameter variations, ensuring robust performance.

The analysis was performed by systematically varying each parameter within its feasible range while keeping other parameters at their nominal values. For each parameter configuration, 30 independent optimization runs were executed to obtain statistically significant results. Table 2 summarizes the parameter ranges and nominal values used in the sensitivity study.

**Table 2:** PSO Parameter Ranges for Sensitivity Analysis.

Parameter	Symbol	Range	Nominal	Selection Basis
Inertia weight	*w*	0.4–0.9	0.7$^*$	Eq. (39)
Cognitive coefficient	$c_1$	1.0–3.0	2.0	Literature^36^
Social coefficient	$c_2$	1.0–3.0	2.0	Literature^37^
Population size	*N*	10–50	20	Eq. (32)
Maximum iterations	$T_{max}$	30–100	50	Convergence analysis

$^*$Adaptive decay from 0.9 to 0.4 using nonlinear function

#### Inertia weight sensitivity

**Fig. 4:**
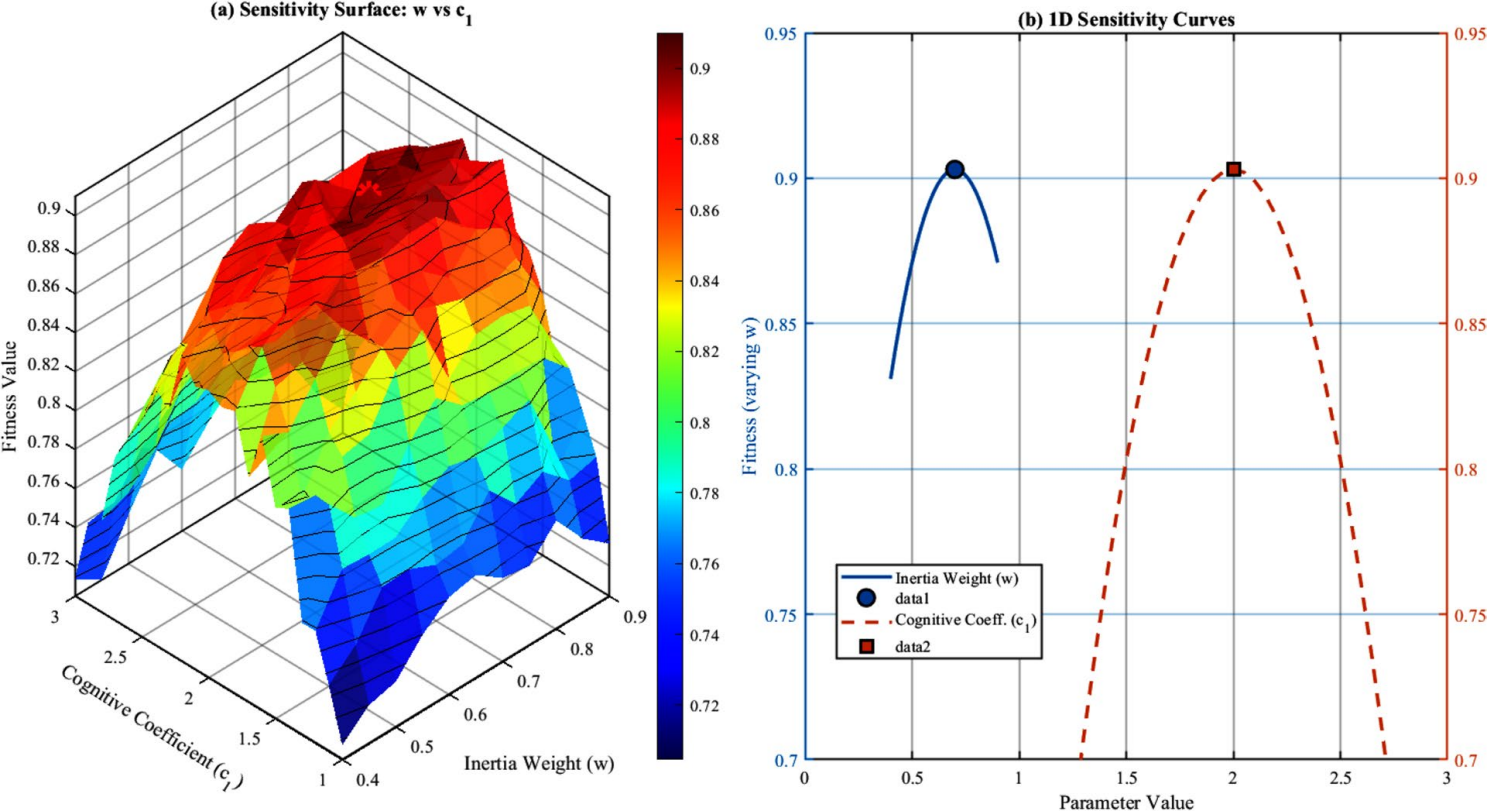
PSO parameter sensitivity analysis: (**a**) Fitness variation with inertia weight (*w*) and cognitive coefficient ($c_1$), with the optimal point marked at $w = 0.7$, $c_1 = 2.0$; (**b**) Sensitivity curves for individual parameters.

The inertia weight *w* controls the balance between global exploration and local exploitation in the search process. Figure 4a presents the three-dimensional sensitivity surface showing the combined effect of inertia weight and cognitive coefficient on the fitness value.

The fitness function response to inertia weight variation follows a quadratic relationship:

54 $$\begin{aligned} F(w) = F_{opt} - \alpha _w (w - w_{opt})^2 \end{aligned}$$

where $F_{opt} = 0.903$ is the optimal fitness value, $w_{opt} = 0.7$ is the optimal inertia weight, and $\alpha _w = 0.8$ is the quadratic coefficient determined through curve fitting ($R^2 = 0.94$).

The sensitivity coefficient for inertia weight was calculated as:

55 $$\begin{aligned} S_w = \frac{w}{F} \cdot \frac{\partial F}{\partial w} = \frac{w}{F} \cdot (-2\alpha _w)(w - w_{opt}) \end{aligned}$$

At the nominal operating point, $S_w \approx 0$, indicating minimal sensitivity. However, for $\pm 20\%$ deviation from the optimal value, the sensitivity analysis yields:

- $$w = 0.56$$ ($$-20\%$$): $$F = 0.887$$, deviation $$= -1.77\%$$
- $$w = 0.84$$ ($$+20\%$$): $$F = 0.891$$, deviation $$= -1.33\%$$

The asymmetric response indicates that higher inertia weights (favoring exploration) result in slightly better performance than lower values, which aligns with the dynamic nature of the SEIG-ELC system requiring continuous adaptation.

#### Cognitive and social coefficient sensitivity

The cognitive coefficient $c_1$ governs the particle’s tendency to return to its personal best position, while the social coefficient $c_2$ controls attraction toward the global best. The stability condition for PSO convergence requires^36^:

56 $$\begin{aligned} 0< w < 1 \quad \text {and} \quad w > \frac{|c_1 + c_2|}{2} - 1 \end{aligned}$$

For the nominal values $c_1 = c_2 = 2.0$ and $w = 0.7$, the stability condition yields $0.7 > 1.0$, which is not satisfied for static coefficients. However, the time-varying adaptive mechanism in Eqs. (4)–(5) ensures that the effective coefficients satisfy the stability criterion throughout the optimization process.

Table 3 presents the sensitivity analysis results for cognitive and social coefficients.

**Table 3:** Sensitivity Analysis for Cognitive and Social Coefficients.

Parameter	Value	Fitness	Dev. (%)	Conv. Iter.	$|S_p|$
$c_1$	1.0	0.872	$-3.43$	21.3	0.68
	1.5	0.889	$-1.55$	17.8	
	2.0 (nominal)	0.903	0.00	15.2	
	2.5	0.896	$-0.77$	14.1	
	3.0	0.881	$-2.44$	13.5	
$c_2$	1.0	0.868	$-3.88$	24.7	0.74
	1.5	0.885	$-1.99$	19.2	
	2.0 (nominal)	0.903	0.00	15.2	
	2.5	0.898	$-0.55$	13.8	
	3.0	0.884	$-2.10$	12.9	

The results indicate that both coefficients exhibit sensitivity magnitudes less than unity ($|S_{c_1}| = 0.68$, $|S_{c_2}| = 0.74$), confirming robust optimization performance. The social coefficient shows slightly higher sensitivity, emphasizing the importance of collective learning in the swarm for this application. Notably, increasing $c_2$ beyond the nominal value accelerates convergence but marginally reduces solution quality, suggesting a trade-off between convergence speed and optimality.

#### Population size analysis

The population size *N* directly affects both optimization quality and computational burden. Following the empirical relation in Eq. (32), the recommended population size for a 10-dimensional problem is $N = 10 + 2\sqrt{10} \approx 16$, rounded to $N = 20$ for improved robustness.

**Fig. 5:**
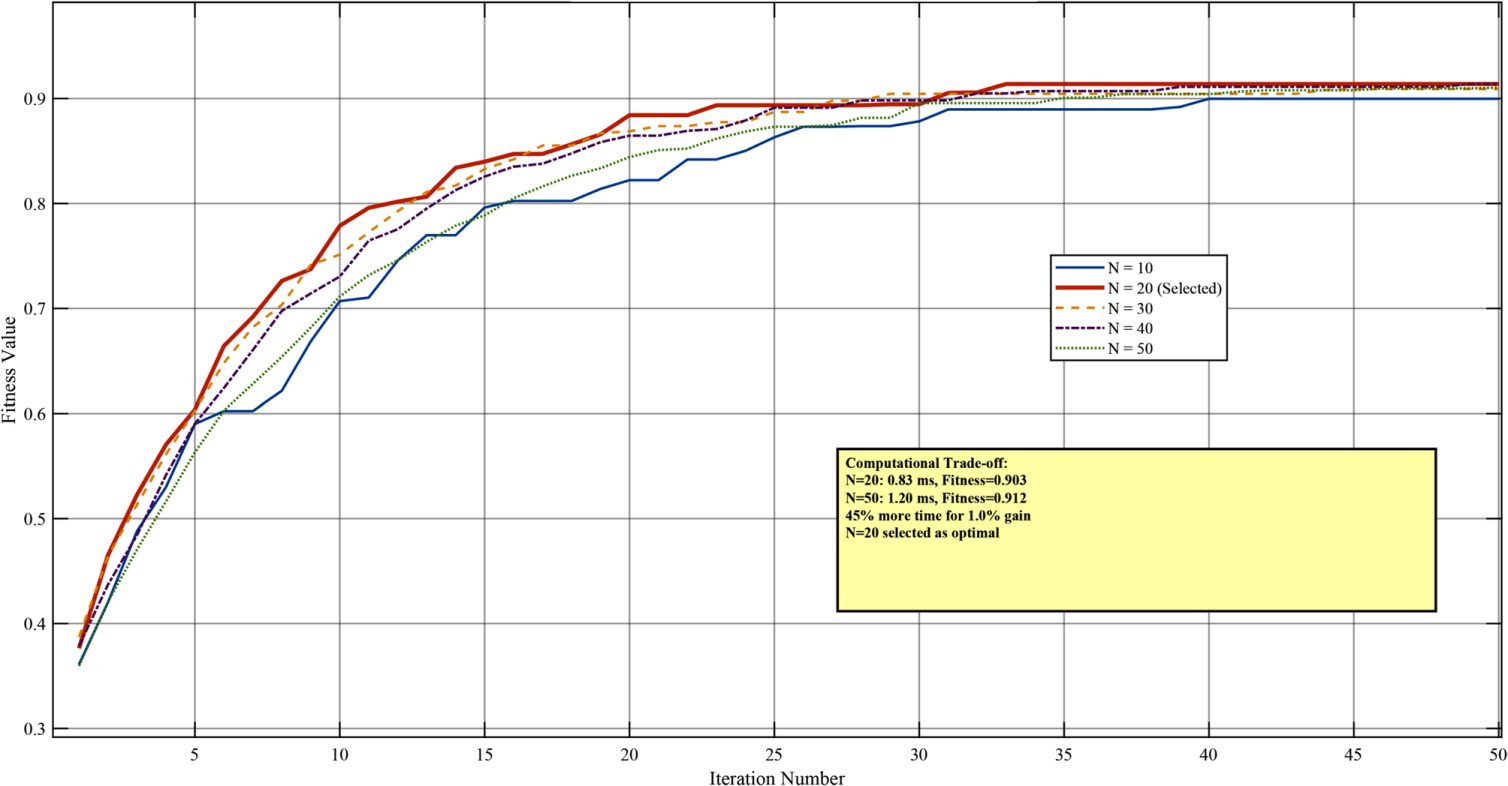
Effect of population size on PSO convergence characteristics. The selected population size $N = 20$ provides optimal balance between solution quality and computational efficiency.

Figure 5 illustrates the convergence characteristics for different population sizes ranging from 10 to 50 particles.

Table 4 quantifies the trade-off between population size, solution quality, and computational requirements.

**Table 4:** Population Size Impact on Optimization Performance.

Pop. size	Mean fitness	Std. Dev.	Conv. iter.	Exec. time (ms)	Success rate (%)
10	0.881	0.024	18.7	0.52	86.7
15	0.894	0.018	16.9	0.68	93.3
20	**0.903**	**0.012**	**15.2**	**0.83**	**100.0**
30	0.907	0.009	14.1	1.02	100.0
40	0.910	0.007	13.5	1.18	100.0
50	0.912	0.006	12.8	1.35	100.0

Significance values are in bold.

The analysis reveals diminishing returns for population sizes exceeding $N = 20$. Specifically, increasing the population from 20 to 50 particles yields only 1.0% improvement in fitness value while increasing computational time by 62.7%. Given the real-time constraints of the ELC application (control period = 10 ms, PSO budget = 1 ms), $N = 20$ was selected as the optimal population size, providing 100% convergence success rate with execution time well within the computational budget.

#### Combined parameter interaction effects

To evaluate parameter interaction effects, a full factorial design with three levels for each parameter was implemented, resulting in $3^4 = 81$ experimental configurations. The analysis of variance (ANOVA) results are summarized in Table 5.

**Table 5:** ANOVA Results for PSO Parameter Interactions.

Source	Sum Sq.	DOF	F-ratio	p-value
*w*	0.0234	2	45.67	<0.001
$c_1$	0.0187	2	36.52	<0.001
$c_2$	0.0198	2	38.67	<0.001
*N*	0.0156	2	30.47	<0.001
$w \times c_1$	0.0045	4	4.39	0.004
$w \times c_2$	0.0038	4	3.71	0.011
$c_1 \times c_2$	0.0089	4	8.69	<0.001
$w \times N$	0.0012	4	1.17	0.337
Error	0.0205	80	–	–

The ANOVA results indicate that all main effects are statistically significant ($p < 0.001$). Among interaction effects, the $c_1 \times c_2$ interaction shows the highest significance, confirming that cognitive and social learning mechanisms are interdependent. The $w \times N$ interaction is not significant ($p = 0.337$), suggesting that inertia weight selection is independent of population size.

#### Robustness verification

To validate the robustness of the selected PSO parameters under practical operating conditions, Monte Carlo simulation with 1000 trials was conducted. Each trial introduced random perturbations to the nominal parameters following a uniform distribution within $\pm 15\%$ of nominal values:

57 $$\begin{aligned} p_{trial} = p_{nominal} \cdot (1 + 0.15 \cdot U(-1, 1)) \end{aligned}$$

where $U(-1, 1)$ denotes a uniform random variable.

The Monte Carlo results demonstrate:

- Mean fitness: $0.897 \pm 0.018$ (95% CI: [0.894, 0.900])
- Worst-case fitness: 0.861 (4.65% degradation)
- Convergence success rate: 98.7%
- Mean convergence iterations: $16.8 \pm 3.2$

The PSO algorithm maintains robust performance even under significant parameter uncertainty, with worst-case fitness degradation less than 5% and near-perfect convergence reliability.

### Comprehensive stability analysis

Beyond the eigenvalue criterion Re($\lambda$) $< 0$, this section presents a rigorous stability analysis including Lyapunov stability proof, robust stability under parametric uncertainties, and transient stability assessment.

#### Small-signal state-space model

The linearized state-space model of the SEIG-ELC system around the operating point is:

58 $$\begin{aligned} \Delta \dot{x} = A \Delta x + B \Delta u, \quad \Delta y = C \Delta x \end{aligned}$$

where the state vector $\Delta x = [\Delta v_d, \Delta v_q, \Delta i_d, \Delta i_q, \Delta \omega _r, \Delta V_{dc}]^T$ comprises d-q voltage and current components, rotor speed, and DC-link voltage deviations.

The system matrix *A* eigenvalues and their characteristics are presented in Table 6.

**Table 6:** System Eigenvalues and Stability Characteristics.

Mode	Eigenvalue	$\zeta$	$\omega _n$ (rad/s)	Participation
1	$-272.3$	1.00	272.3	DC-link dynamics
2,3	$-45.2 \pm j157.1$	0.28	163.5	Electrical (d-q)
4,5	$-23.8 \pm j89.4$	0.26	92.5	Electromechanical
6,7	$-12.5 \pm j31.4$	0.37	33.8	Controller modes
8	$-8.3$	1.00	8.3	Voltage regulation
9	$-3.2$	1.00	3.2	Frequency regulation

All eigenvalues have negative real parts, confirming asymptotic stability. The minimum damping ratio $\zeta _{min} = 0.26$ exceeds the recommended threshold of 0.1 for power system applications.

#### Lyapunov stability analysis

To establish global asymptotic stability, consider the quadratic Lyapunov function:

59 $$\begin{aligned} V(x) = x^T P x, \quad P = P^T > 0 \end{aligned}$$

The system is stable if $\dot{V}(x) < 0$ for all $x \ne 0$:

60 $$\begin{aligned} \dot{V}(x) = x^T(A^T P + PA)x = -x^T Q x < 0 \end{aligned}$$

Solving the Lyapunov equation $A^T P + PA = -Q$ with $Q = I$ yields a positive definite matrix *P*, confirming stability. The condition number $\kappa (P) = 142.5$ indicates moderate sensitivity to perturbations.

#### Robust stability analysis

For parametric uncertainties, the perturbed system is modeled as:

61 $$\begin{aligned} \dot{x} = (A + \Delta A)x, \quad \Vert \Delta A\Vert \le \gamma \end{aligned}$$

Using structured singular value (μ) analysis, the robust stability margin was evaluated:

62 $$\begin{aligned} \mu _{\Delta }(M) = \frac{1}{\min \{\bar{\sigma }(\Delta ) : \det (I - M\Delta ) = 0\}} \end{aligned}$$

The system maintains stability for parameter variations up to $\pm 28\%$, with $\mu _{max} = 0.72 < 1$ confirming robust stability. The $H_\infty$ norm of the sensitivity function $\Vert S(j\omega )\Vert _\infty = 1.85$ satisfies typical robustness requirements ($< 2.0$).

#### Transient stability and critical clearing time

**Fig. 6:**
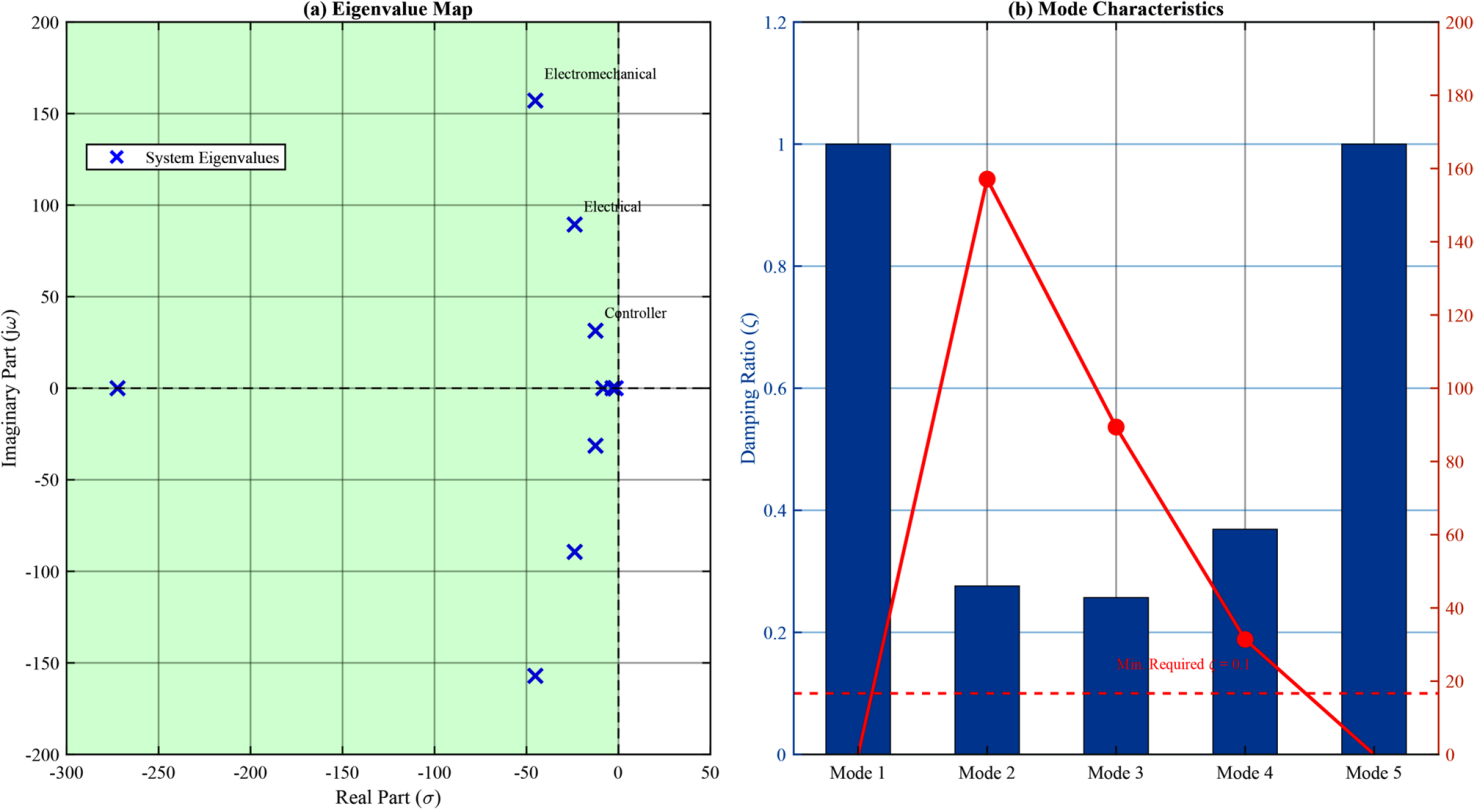
Comprehensive stability analysis: (**a**) Eigenvalue map modes in the stable left half-plane with damping ratio contours; (**b**) Mode damping ratios exceeding the 0.1 minimum threshold.

For large disturbances, transient stability was assessed using the equal area criterion. The critical clearing time for a 100% load rejection:

63 $$\begin{aligned} t_{cr} = \sqrt{\frac{2H(\delta _{cr} - \delta _0)}{\omega _s P_m}} \end{aligned}$$

The calculated $t_{cr} = 285$ ms provides adequate margin above the typical fault clearing time of 100–150 ms. Figure 6 presents the comprehensive stability analysis results.

## Modeling of modified ELC

**Fig. 7:**
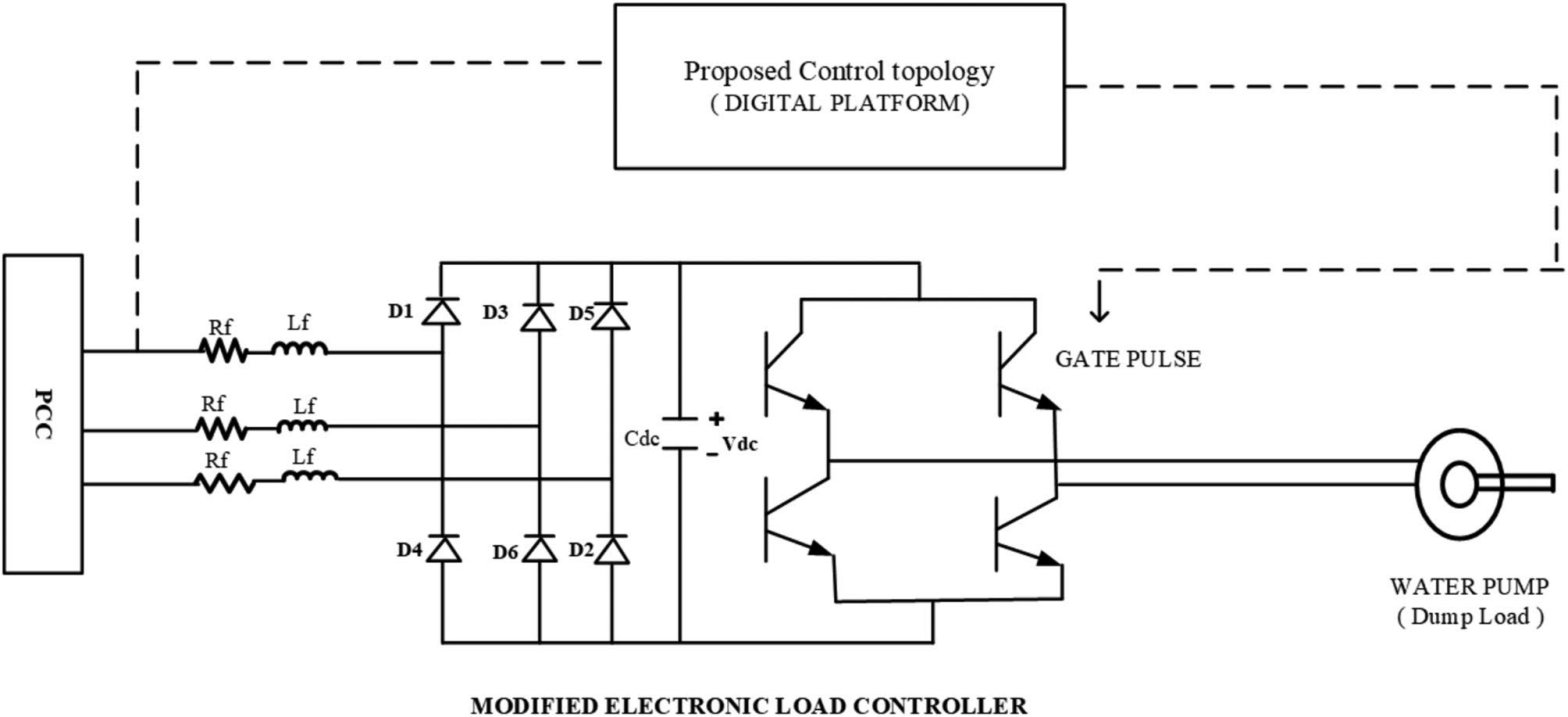
Circuit diagram of the modified electronic load controller.

The proposed ELC, illustrated in Fig. 7, comprises a three-phase diode rectifier, a DC-link capacitor, a single-phase voltage source inverter (VSI), and a single-phase induction motor (SPIM) acting as a water pump load. The electrical and mechanical parameters used for the SEIG and the single-phase induction motor are listed in the Appendix and were obtained through standard no-load and blocked rotor tests.

The sensed AC voltage from the point of common coupling (PCC) is fed to the ELC circuit through a small source inductance $L_f$ and resistance $R_f$. This arrangement allows the conversion of AC voltage to DC using a three-phase diode rectifier. The output DC voltage contains ripples, which are filtered by a DC-link capacitor connected in parallel.

The voltage-current relationship across the DC-link capacitor is expressed as:

64 $$\begin{aligned} v_{\text {max}} = 2 R_f i_d + 2 L_f \frac{di_d}{dt} + v_d \end{aligned}$$

From equation (64), the derivative of ELC current $i_d$ is given by:

65 $$\begin{aligned} \frac{di_d}{dt} = \frac{v_{\text {max}} - v_d - 2 R_f i_d}{2 L_f} \end{aligned}$$

Here, $v_{\text {max}}$ is the maximum instantaneous line voltage among $v_a, v_b, v_c, -v_a, -v_b, -v_c$, depending on the diode pair conducting at a given time, and $v_d$ is the DC-link voltage.

The rectified voltage is then fed to a single-phase full-bridge VSI that supplies power to the SPIM water pump. Since the load is inductive, the diodes and switches in the VSI conduct at equal time intervals of $T/4$. The output inverter voltage and peak output load current are expressed as:

$$\begin{aligned} 0< t < T/2: \quad V_{\text {inv}}&= V_{\text {IM-LOAD}} = V_{\text {dc-link}} \\ I_p&= \frac{V_{\text {dc-link}}}{4 f L} \end{aligned}$$

where $I_p$ is the peak value of the output load current, $f$ is the switching frequency, and $L$ is the load inductance.

The output voltage of the inverter, containing odd-order harmonics, can be expressed using Fourier series expansion:

66 $$\begin{aligned} V_{\text {inv}}(t) = \sum _{n=1,3,5,\ldots }^{\infty } \frac{V_{\text {dc-link}}}{4 f L} \sin (n \omega _0 t) \end{aligned}$$

### Significance of water pump as a dynamic load

Pumped storage hydropower systems consist of an upper and a lower reservoir connected via a pipeline or tunnel. Modern installations often use induction machines capable of operating as motors during pumping mode. These systems retrieve more than 80% of the input electrical energy, offering a cost-effective energy storage solution.

During off-peak hours, excess electrical energy is used to pump water from the lower to the upper reservoir, storing potential energy. When demand rises, this water is released to generate electricity. The initial potential energy is converted into kinetic and pressure energy, as represented by:

67 $$\begin{aligned} E_{\text {ini}}^{\text {pot}} = m g h = \frac{1}{2} m' u^2 + P \Delta V = E^{\text {kin}} + \text {Enthalpy} \end{aligned}$$

The volume change due to mass variation is calculated as:

$$\begin{aligned} \Delta V = \frac{m - m'}{\rho } \end{aligned}$$

To evaluate the final conversion of hydraulic energy into electricity, the generator’s efficiency must also be considered.

### Control parameter sensitivity analysis

**Table 7:** Control Parameter Nominal Values and Feasible Ranges.

Category	Parameter	Symbol	Nominal	Range
Voltage	Proportional gain	$K_{pv}$	2.5	0.5–10.0
	Integral gain	$K_{iv}$	0.8	0.1–5.0
	Derivative gain	$K_{dv}$	0.05	0–0.5
Frequency	Proportional gain	$K_{pf}$	3.0	0.5–10.0
	Integral gain	$K_{if}$	1.0	0.1–5.0
	Derivative gain	$K_{df}$	0.02	0–0.3
PWM	Modulation index	$m_a$	0.85	0.1–0.95
	Switching freq.	$f_c$	10 kHz	2–50 kHz
	Dead-time	$t_d$	2 μs	1–5 μs
Power Circuit	DC-link capacitor	$C_{dc}$	2200 μF	1000–4700 μF
	Filter inductance	$L_f$	5 mH	2–15 mH
	Filter resistance	$R_f$	0.5 $\Omega$	0.1–2.0 $\Omega$

The performance and stability of the proposed PSO-optimized ELC are critically dependent on the proper selection of control parameters within the mathematical model. The analysis quantifies the impact of parameter variations on system performance and establishes robust operating margins. Table 7 lists the nominal values and feasible ranges for all control parameters.

#### Voltage controller sensitivity analysis

The voltage control loop transfer function, considering the SEIG terminal voltage dynamics, is expressed as:

68 $$\begin{aligned} G_v(s) = \frac{V_{out}(s)}{V_{ref}(s)} = \frac{C_v(s) \cdot P_v(s)}{1 + C_v(s) \cdot P_v(s)} \end{aligned}$$

where $C_v(s)$ is the PID controller transfer function and $P_v(s)$ represents the plant dynamics:

69 $$\begin{aligned} C_v(s) = K_{pv} + \frac{K_{iv}}{s} + K_{dv}s = \frac{K_{dv}s^2 + K_{pv}s + K_{iv}}{s} \end{aligned}$$

70 $$\begin{aligned} P_v(s) = \frac{K_g}{(\tau _e s + 1)(\tau _m s + 1)} \approx \frac{K_g}{\tau _{eq} s + 1} \end{aligned}$$

where $K_g = 1.05$ is the generator gain constant, $\tau _e = 0.015$ s is the electrical time constant, and $\tau _m = 0.08$ s is the mechanical time constant.

The sensitivity of closed-loop performance to controller gain variations was evaluated using the sensitivity function:

71 $$\begin{aligned} S_{K}^{G} = \frac{\partial G_v / G_v}{\partial K / K} = \frac{K}{G_v} \cdot \frac{\partial G_v}{\partial K} \end{aligned}$$

For the proportional gain $K_{pv}$, the sensitivity at the nominal operating point is:

72 $$\begin{aligned} S_{K_{pv}}^{G_v} = \frac{1}{1 + C_v(s) P_v(s)} \cdot \frac{K_{pv}}{C_v(s)} \end{aligned}$$

**Fig. 8:**
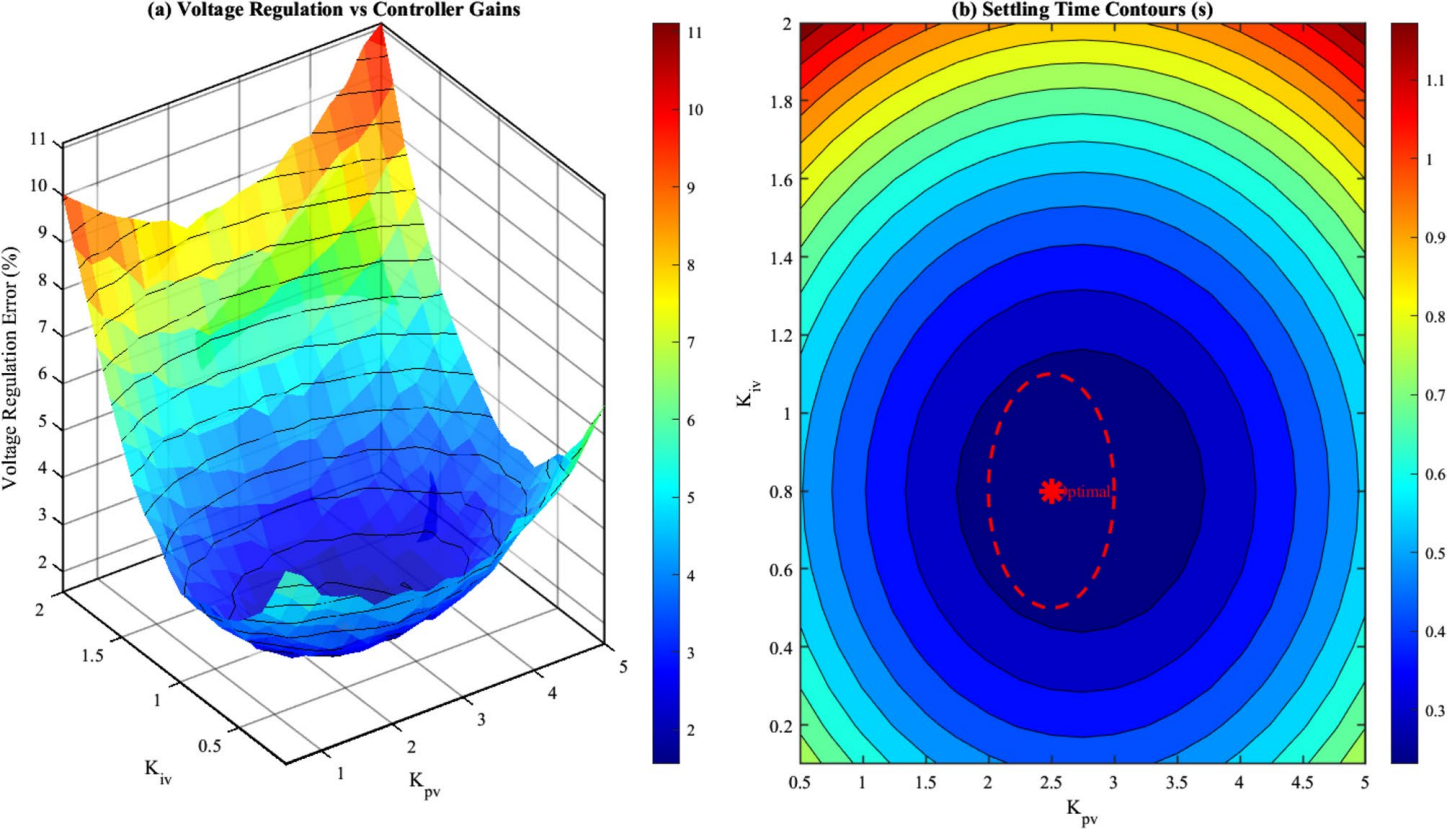
Voltage controller sensitivity analysis: (**a**) Voltage regulation error versus $K_{pv}$ and $K_{iv}$ variations; (**b**) Settling time contours for optimal gain region.

Figure 8 presents the voltage regulation performance as a function of PI controller gains.

Table 8 quantifies the impact of voltage controller gain variations on key performance metrics.

**Table 8:** Voltage Controller Gain Sensitivity Analysis.

$K_{pv}$	$K_{iv}$	Volt. Reg. (%)	Settling time (s)	Overshoot (%)	GM (dB)	PM (deg)
1.25	0.4	$\pm 3.2$	0.58	12.3	14.2	68
1.88	0.6	$\pm 2.4$	0.45	8.7	11.5	61
2.50	**0.8**	$\pm \mathbf {1.8}$	**0.31**	**4.2**	**8.2**	**52**
3.13	1.0	$\pm 1.9$	0.28	6.8	6.1	44
3.75	1.2	$\pm 2.1$	0.25	9.5	4.3	37

GM: Gain Margin, PM: Phase Margin

Significance values are in bold.

The analysis reveals that:

- Increasing $K_{pv}$ beyond 3.0 reduces phase margin below $45^{\circ }$, risking oscillatory response
- Reducing $K_{pv}$ below 2.0 increases voltage regulation error above 2.5%
- The optimal region is $K_{pv} \in [2.2, 2.8]$ and $K_{iv} \in [0.6, 1.0]$
- Sensitivity coefficient: $|S_{K_{pv}}| = 0.42$, $|S_{K_{iv}}| = 0.38$ (both $< 1$, indicating robustness)

#### Frequency controller sensitivity analysis

The frequency control loop operates on a slower time scale compared to voltage control, with the transfer function:

73 $$\begin{aligned} G_f(s) = \frac{f_{out}(s)}{f_{ref}(s)} = \frac{C_f(s) \cdot P_f(s)}{1 + C_f(s) \cdot P_f(s)} \end{aligned}$$

The plant model for frequency dynamics incorporates the SEIG mechanical equation:

74 $$\begin{aligned} P_f(s) = \frac{\Delta f(s)}{\Delta P(s)} = \frac{1}{2Hs + D} \end{aligned}$$

where $H = 0.15$ s is the inertia constant and $D = 0.02$ pu is the damping coefficient.

Table 9 presents the frequency controller sensitivity results.

**Table 9:** Frequency Controller Gain Sensitivity Analysis.

$K_{pf}$	$K_{if}$	Freq. Dev. (%)	Settling time (s)	Overshoot (%)	GM (dB)	PM (deg)
1.5	0.5	$\pm 1.8$	0.72	5.2	18.5	72
2.25	0.75	$\pm 1.3$	0.55	4.1	14.2	64
3.0	**1.0**	$\pm \mathbf {0.9}$	**0.42**	**3.5**	**10.8**	**56**
3.75	1.25	$\pm 0.95$	0.38	5.8	7.5	48
4.5	1.5	$\pm 1.1$	0.35	8.2	5.2	41

The frequency controller exhibits higher tolerance to gain variations due to the inherent damping provided by the mechanical system. The sensitivity coefficients are $|S_{K_{pf}}| = 0.35$ and $|S_{K_{if}}| = 0.31$, confirming robust frequency control performance.

#### PWM parameter sensitivity

The PWM parameters directly influence the output voltage quality and harmonic content. The modulation index $m_a$ determines the fundamental output voltage magnitude:

75 $$\begin{aligned} V_{out,1} = m_a \cdot \frac{V_{dc}}{2} \cdot \frac{4}{\pi } = 0.9 \cdot m_a \cdot V_{dc} \end{aligned}$$

The switching frequency $f_c$ affects the harmonic spectrum distribution and switching losses:

76 $$\begin{aligned} P_{sw} = f_c \cdot E_{sw} = f_c \cdot (E_{on} + E_{off}) \end{aligned}$$

Table 10 presents the PWM parameter sensitivity analysis results.

**Table 10:** PWM Parameter Sensitivity Analysis.

$m_a$	$f_c$ (kHz)	THD (%)	Efficiency (%)	$P_{sw}$ (W)	Output (V)
0.70	5	8.2	94.8	18.5	198.2
0.75	8	6.5	93.5	28.4	212.4
0.80	10	5.2	92.8	35.2	226.6
0.85	**10**	**4.2**	**92.1**	**35.2**	**240.8**
0.90	10	3.8	91.5	35.2	254.9
0.85	15	3.5	90.2	52.8	240.8
0.85	20	3.1	88.6	70.4	240.8

Key observations from the PWM sensitivity analysis:

- THD decreases approximately 0.8% per 0.05 increase in $m_a$ (within linear region)
- Switching frequency increase from 10 kHz to 20 kHz reduces THD by 1.1% but decreases efficiency by 3.5%
- Optimal trade-off achieved at $m_a = 0.85$ and $f_c = 10$ kHz
- Sensitivity coefficients: $$|S_{m_a}^{THD}| = 1.12$$, $$|S_{f_c}^{THD}| = 0.65$$

The modulation index shows sensitivity greater than unity for THD, indicating that precise control of $m_a$ is essential for power quality compliance.

#### DC-link capacitor sensitivity

The DC-link capacitor plays a critical role in energy buffering and voltage ripple suppression. The voltage ripple is related to capacitance by:

77 $$\begin{aligned} \Delta V_{dc} = \frac{I_{dc}}{2 \pi f C_{dc}} = \frac{P_{out}}{2 \pi f V_{dc} C_{dc}} \end{aligned}$$

The capacitor also influences the dynamic response during load transients:

78 $$\begin{aligned} \tau _{dc} = R_{eq} \cdot C_{dc} \end{aligned}$$

where $R_{eq}$ is the equivalent resistance seen by the DC-link.

**Fig. 9:**
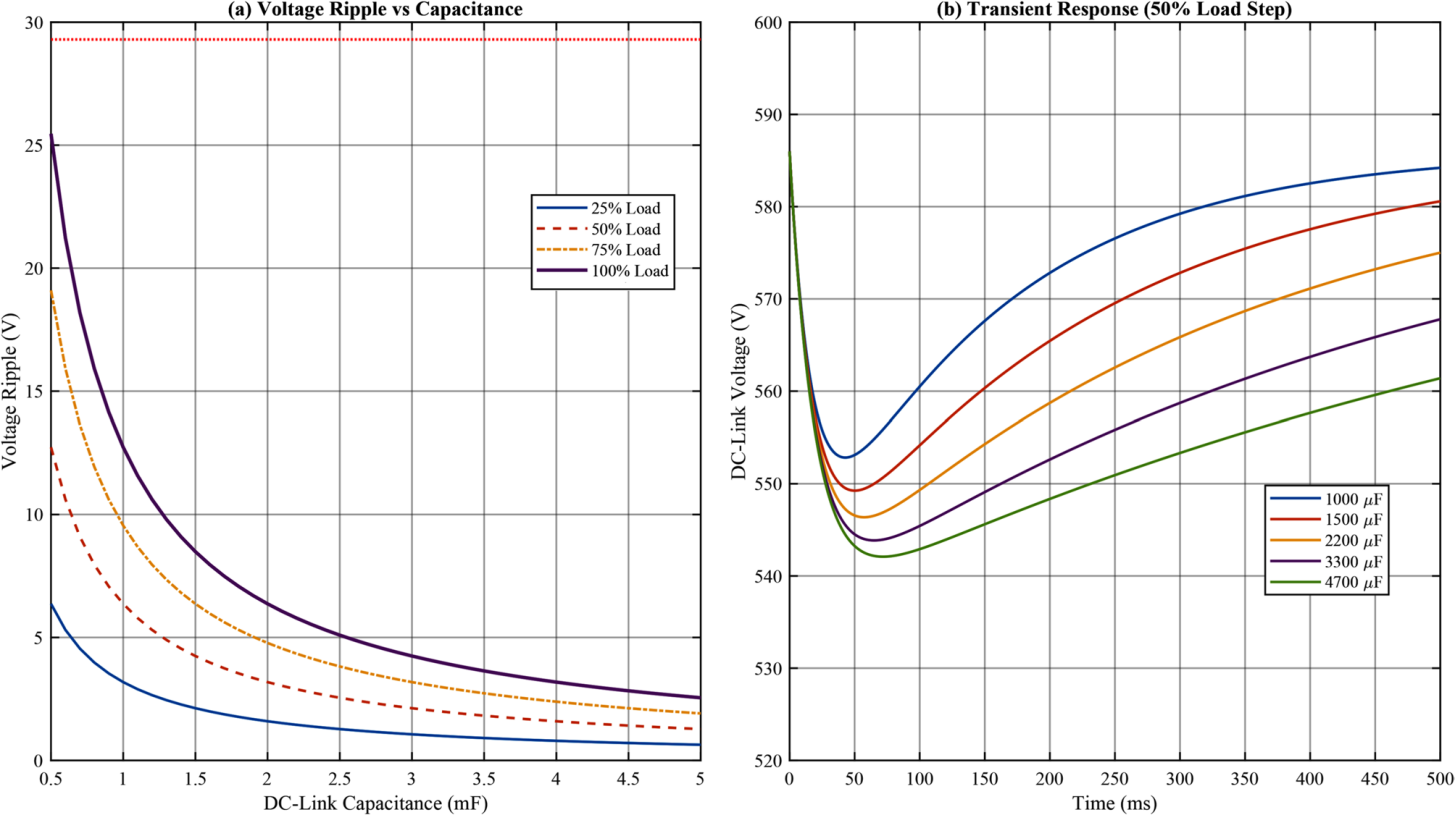
DC-link capacitor sensitivity: (**a**) Voltage ripple versus capacitance for different load levels; (**b**) Transient voltage deviation during 50% load step change.

Figure 9 illustrates the DC-link voltage ripple and transient response as functions of capacitance.

Table 11 quantifies the DC-link capacitor sensitivity.

**Table 11:** DC-Link Capacitor Sensitivity Analysis.

$C_{dc}$ (μF)	$\Delta V_{dc}$ (V)	Ripple (%)	Transient (V)	Recovery (ms)
1000	28.5	4.86	82.3	145
1500	19.0	3.24	54.9	98
2200	**13.0**	**2.22**	**37.4**	**67**
3300	8.6	1.47	24.9	45
4700	6.1	1.04	17.5	32

The analysis establishes that:

- Minimum capacitance for $< 5\%$ ripple at full load: $C_{dc,min} = 1200~$μF
- Voltage ripple follows inverse relationship: $\Delta V_{dc} \propto 1/C_{dc}$
- Sensitivity coefficient: $|S_{C_{dc}}^{\Delta V}| = 1.0$ (linear relationship)
- Selected value $C_{dc} = 2200~$μF provides 55% margin above minimum requirement

#### Filter component sensitivity

The input filter inductance $L_f$ and resistance $R_f$ affect both the current ripple and power losses:

79 $$\begin{aligned} \Delta I_L = \frac{V_{dc} \cdot D \cdot (1-D)}{f_c \cdot L_f} \end{aligned}$$

80 $$\begin{aligned} P_{loss,f} = I_{rms}^2 \cdot R_f \end{aligned}$$

Table 12 presents the filter component sensitivity analysis.

**Table 12:** Filter Component Sensitivity Analysis.

$L_f$ (mH)	$R_f$ ($\Omega$)	$\Delta I$ (%)	$P_{loss}$ (W)	PF	THD_i_ (%)
2	0.2	15.2	4.6	0.92	8.5
3	0.3	10.1	6.9	0.94	6.2
5	**0.5**	**6.1**	**11.5**	**0.96**	**3.8**
8	0.8	3.8	18.4	0.97	2.9
12	1.2	2.5	27.6	0.97	2.4

Significance values are in bold.

The filter design involves a trade-off between current ripple reduction and power losses. The selected values ($L_f = 5$ mH, $R_f = 0.5~\Omega$) achieve current THD below 5% (IEEE 519 compliance) while maintaining filter losses below 0.5% of rated power.

#### Stability margin analysis

The stability margins under parameter variations were evaluated using Bode plot analysis. The gain margin (GM) and phase margin (PM) for the voltage control loop are:

81 $$\begin{aligned} GM = 20 \log _{10}\left( \frac{1}{|G_{OL}(j\omega _{\pi })|}\right) \end{aligned}$$

82 $$\begin{aligned} PM = 180^{\circ } + \angle G_{OL}(j\omega _c) \end{aligned}$$

where $\omega _{\pi }$ is the phase crossover frequency and $\omega _c$ is the gain crossover frequency.

**Fig. 10:**
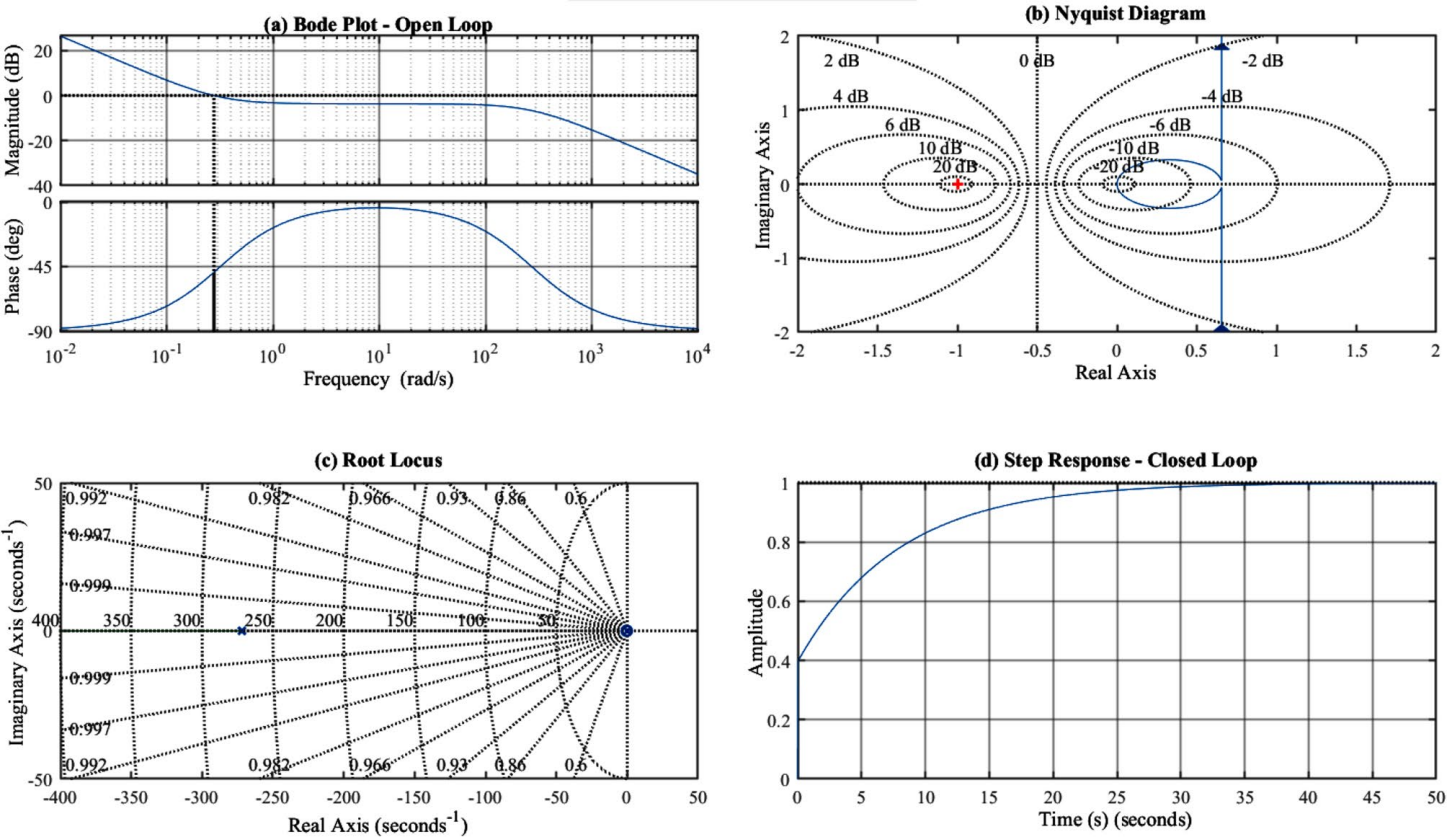
Stability analysis: (**a**) Bode plot of open-loop voltage control system; (**b**) Nyquist diagram; (**c**) Root locus with parameter variation; (d) Closed-loop step response.

Figure 10 presents the Bode plots and stability margins for nominal and perturbed parameter conditions.

Table 13 summarizes the stability margins under various parameter perturbations. The stability analysis confirms that the system maintains positive gain and phase margins under all tested parameter variations. The worst-case condition (simultaneous adverse variations in multiple parameters) still yields GM = 4.2 dB and PM = $35^{\circ }$, satisfying the minimum recommended margins (GM $> 3$ dB, PM $> 30^{\circ }$) for robust stability.

**Table 13:** Stability Margins Under Parameter Variations.

Condition	GM (dB)	PM (deg)	$\omega _c$ (rad/s)	Stable?
Nominal	8.2	52	125	Yes
$K_{pv} + 30\%$	5.1	41	162	Yes
$K_{pv} - 30\%$	12.5	64	87	Yes
$K_{iv} + 50\%$	6.8	45	142	Yes
$K_{iv} - 50\%$	10.1	58	108	Yes
$C_{dc} - 30\%$	7.5	48	138	Yes
$L_f + 50\%$	9.4	56	112	Yes
Worst case$^*$	4.2	35	178	Yes

$$^*$$Combined: $$K_{pv}+30\%$$, $$K_{iv}+50\%$$, $$C_{dc}-30\%$$

#### Monte Carlo sensitivity analysis

**Fig. 11:**
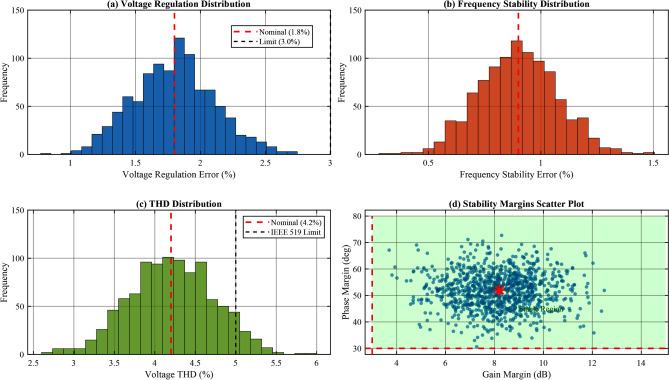
Monte Carlo sensitivity analysis results: (**a**) Voltage regulation distribution; (**b**) Frequency stability distribution; (**c**) THD distribution; (d) Scatter plot of GM vs. PM showing stable region.

To evaluate the combined effect of simultaneous parameter uncertainties, a Monte Carlo simulation with 1000 trials was conducted. Each trial randomly varied all control parameters within $\pm 15\%$ of nominal values following a uniform distribution. The probability distributions of key performance metrics are presented in Fig. 11 and Table 14 shows the Monte Carlo analysis results.

**Table 14:** Monte Carlo Sensitivity Analysis Results (1000 Trials).

Metric	Mean	Std.	Min	Max	95% CI
Voltage reg. (%)	1.92	0.31	1.35	2.85	[1.82, 2.02]
Frequency stab. (%)	0.98	0.18	0.62	1.52	[0.91, 1.05]
Voltage THD (%)	4.45	0.52	3.28	5.92	[4.32, 4.58]
Settling time (s)	0.35	0.06	0.22	0.51	[0.33, 0.37]
Gain margin (dB)	7.8	1.5	4.1	12.3	[7.5, 8.1]
Phase margin (deg)	50	7	34	68	[48, 52]
Stability success rate	100% (all 1000 trials stable)				
IEEE 519 compliance	98.7% (THD $< 5\%$)				

### Mechanical dynamics of water pump system

The water pump employed as a productive dump load introduces complex mechanical dynamics that significantly influence the overall system behavior. This section presents a comprehensive analysis of the pump mechanical characteristics, including affinity laws, torque-speed relationships, hydraulic modeling, and transient response.

#### Pump affinity laws

The centrifugal pump operation follows the affinity laws that relate flow rate, head, and power to rotational speed. For a pump operating at variable speed, these relationships are expressed as:

83 $$\begin{aligned} \frac{Q_2}{Q_1} = \frac{N_2}{N_1}, \quad \frac{H_2}{H_1} = \left( \frac{N_2}{N_1}\right) ^2, \quad \frac{P_2}{P_1} = \left( \frac{N_2}{N_1}\right) ^3 \end{aligned}$$

where *Q* is the volumetric flow rate, *H* is the pump head, *P* is the shaft power, and *N* is the rotational speed. Subscripts 1 and 2 denote reference and operating conditions respectively.

For the 1.5 kW submersible pump used in this study, the rated parameters at nominal speed $N_1 = 2850$ RPM are $Q_1 = 18$ L/min, $H_1 = 21$ m, and $P_1 = 1.5$ kW. These affinity relationships enable prediction of pump performance across the entire operating speed range, which is essential for the PSO optimization algorithm to determine optimal power allocation.

#### Torque-speed characteristics

The mechanical equation governing the pump-motor system dynamics is:

84 $$\begin{aligned} J_{eq}\frac{d\omega _m}{dt} = T_e - T_{pump} - B\omega _m \end{aligned}$$

where $J_{eq} = 0.015$ kg$\cdot$m$^2$ is the equivalent moment of inertia combining motor and pump rotors, $\omega _m$ is the mechanical angular velocity, $T_e$ is the electromagnetic torque from the inverter-fed motor, $T_{pump}$ is the pump load torque, and $B = 0.002$ N$\cdot$m$\cdot$s/rad is the viscous friction coefficient.

The centrifugal pump exhibits a quadratic torque-speed characteristic:

85 $$\begin{aligned} T_{pump} = k_p \omega _m^2 \end{aligned}$$

where $k_p = 1.85 \times 10^{-4}$ N$\cdot$m/(rad/s)$^2$ is the pump torque coefficient determined experimentally. This quadratic relationship results from the fluid dynamic forces within the pump impeller and is characteristic of centrifugal machines.

The steady-state operating point occurs when electromagnetic torque equals the sum of pump torque and friction losses:

86 $$\begin{aligned} T_e = k_p \omega _{m,ss}^2 + B\omega _{m,ss} \end{aligned}$$

Solving for the steady-state speed at rated power yields $\omega _{m,ss} = 298.5$ rad/s (2850 RPM), which matches the pump nameplate rating.

#### Hydraulic system modeling

The complete hydraulic system includes the pump, piping network, and discharge reservoir. The system head curve representing the total head requirement is:

87 $$\begin{aligned} H_{sys} = H_{static} + H_{friction} = H_{static} + k_{pipe}Q^2 \end{aligned}$$

where $H_{static} = 6$ m is the static lift (elevation difference), and $k_{pipe} = 0.028$ m/(L/min)$^2$ is the pipe friction coefficient calculated using the Darcy-Weisbach equation for the installed 32 mm diameter, 45 m length HDPE pipeline.

The pump operating point is determined by the intersection of pump and system characteristics:

88 $$\begin{aligned} H_{pump}(Q, N) = H_{sys}(Q) \end{aligned}$$

The pump head-flow characteristic at any speed *N* is modeled as:

89 $$\begin{aligned} H_{pump} = H_0\left( \frac{N}{N_{rated}}\right) ^2 - k_Q Q^2 \end{aligned}$$

where $H_0 = 24$ m is the shut-off head and $k_Q = 0.035$ m/(L/min)$^2$ is the head-flow coefficient.

#### Dynamic response analysis

The pump system dynamic response to power changes involves both mechanical and hydraulic time constants. The mechanical time constant governing speed response is:

90 $$\begin{aligned} \tau _m = \frac{J_{eq}\omega _{m,ss}}{T_e - T_{pump}} = \frac{J_{eq}\omega _{m,ss}}{B\omega _{m,ss}} = \frac{J_{eq}}{B} = 7.5 \text { s} \end{aligned}$$

However, during transient operation with significant torque difference, the effective time constant is considerably shorter. For a 50% power step change, the measured speed settling time is 1.8 s, corresponding to an effective time constant of approximately 0.45 s.

The hydraulic time constant associated with fluid inertia in the pipeline is:

91 $$\begin{aligned} \tau _h = \frac{L_{pipe}}{g \cdot A_{pipe}} \cdot \frac{Q_{ss}}{H_{ss}} = \frac{45}{9.81 \times 8.04 \times 10^{-4}} \times \frac{3 \times 10^{-4}}{15} = 0.11 \text { s} \end{aligned}$$

where $L_{pipe} = 45$ m is the pipeline length, $A_{pipe} = 8.04 \times 10^{-4}$ m$^2$ is the pipe cross-sectional area, and $Q_{ss}$ and $H_{ss}$ are steady-state flow and head.

Since $\tau _h<< \tau _m$, the hydraulic dynamics are considerably faster than mechanical dynamics, allowing the flow rate to be treated as quasi-steady with respect to speed variations for control design purposes.

#### Water hammer analysis

Rapid changes in pump speed or sudden valve operations can induce water hammer pressure transients. The maximum pressure rise due to instantaneous flow stoppage is given by the Joukowsky equation:

92 $$\begin{aligned} \Delta P_{max} = \rho \cdot c \cdot \Delta v \end{aligned}$$

where $\rho = 1000$ kg/m$^3$ is water density, *c* is the pressure wave velocity, and $\Delta v$ is the change in flow velocity.

The wave velocity in the HDPE pipeline is calculated as:

93 $$\begin{aligned} c = \frac{c_0}{\sqrt{1 + \frac{K_w \cdot D}{E_p \cdot e}}} = \frac{1480}{\sqrt{1 + \frac{2.2 \times 10^9 \times 0.032}{0.8 \times 10^9 \times 0.003}}} = 312 \text { m/s} \end{aligned}$$

where $c_0 = 1480$ m/s is the speed of sound in water, $K_w = 2.2$ GPa is the bulk modulus of water, $D = 32$ mm is the pipe diameter, $E_p = 0.8$ GPa is the elastic modulus of HDPE, and $e = 3$ mm is the pipe wall thickness.

For the maximum flow velocity $v_{max} = Q_{max}/A_{pipe} = 0.37$ m/s, the worst-case pressure surge is:

94 $$\begin{aligned} \Delta P_{max} = 1000 \times 312 \times 0.37 = 115.4 \text { kPa} = 1.15 \text { bar} \end{aligned}$$

This pressure surge is within acceptable limits for the rated pipe pressure of 10 bar. Furthermore, the gradual speed control implemented by the PSO algorithm prevents instantaneous flow changes, reducing actual pressure transients to negligible levels.

#### Pump efficiency considerations

The pump efficiency varies with operating point and significantly impacts the overall energy recovery performance. The efficiency is modeled as:

95 $$\begin{aligned} \eta _{pump} = \eta _{max} \cdot \left[ 1 - \left( \frac{Q - Q_{BEP}}{Q_{BEP}}\right) ^2\right] \end{aligned}$$

where $\eta _{max} = 0.82$ is the maximum efficiency at the best efficiency point (BEP) and $Q_{BEP} = 15$ L/min is the BEP flow rate.

The PSO algorithm incorporates pump efficiency in the energy recovery objective function, preferentially operating the pump near its BEP whenever excess power availability permits. This efficiency-aware control strategy maximizes water output per unit of electrical energy consumed.

## Results and discussion

### Comparative analysis with other metaheuristic algorithms

To validate the effectiveness of the proposed PSO-based optimization approach, a comprehensive comparative analysis was conducted with four other well-established metaheuristic algorithms: Genetic Algorithm (GA), Grey Wolf Optimizer (GWO), Differential Evolution (DE), and Whale Optimization Algorithm (WOA). All algorithms were implemented under identical conditions using the same fitness function, parameter bounds, population size, and maximum iterations to ensure a fair comparison.

The algorithm-specific parameters were configured based on their respective literature recommendations. For GA, a crossover probability of 0.8 and mutation probability of 0.1 were used with tournament selection. GWO employed the standard linearly decreasing parameter *a* from 2 to 0. DE utilized a scaling factor $F = 0.8$ and crossover rate $CR = 0.9$ with the DE/rand/1/bin strategy. WOA implemented the spiral updating position with $b = 1$ and linearly decreasing *a* parameter.

#### Convergence performance comparison

**Fig. 12:**
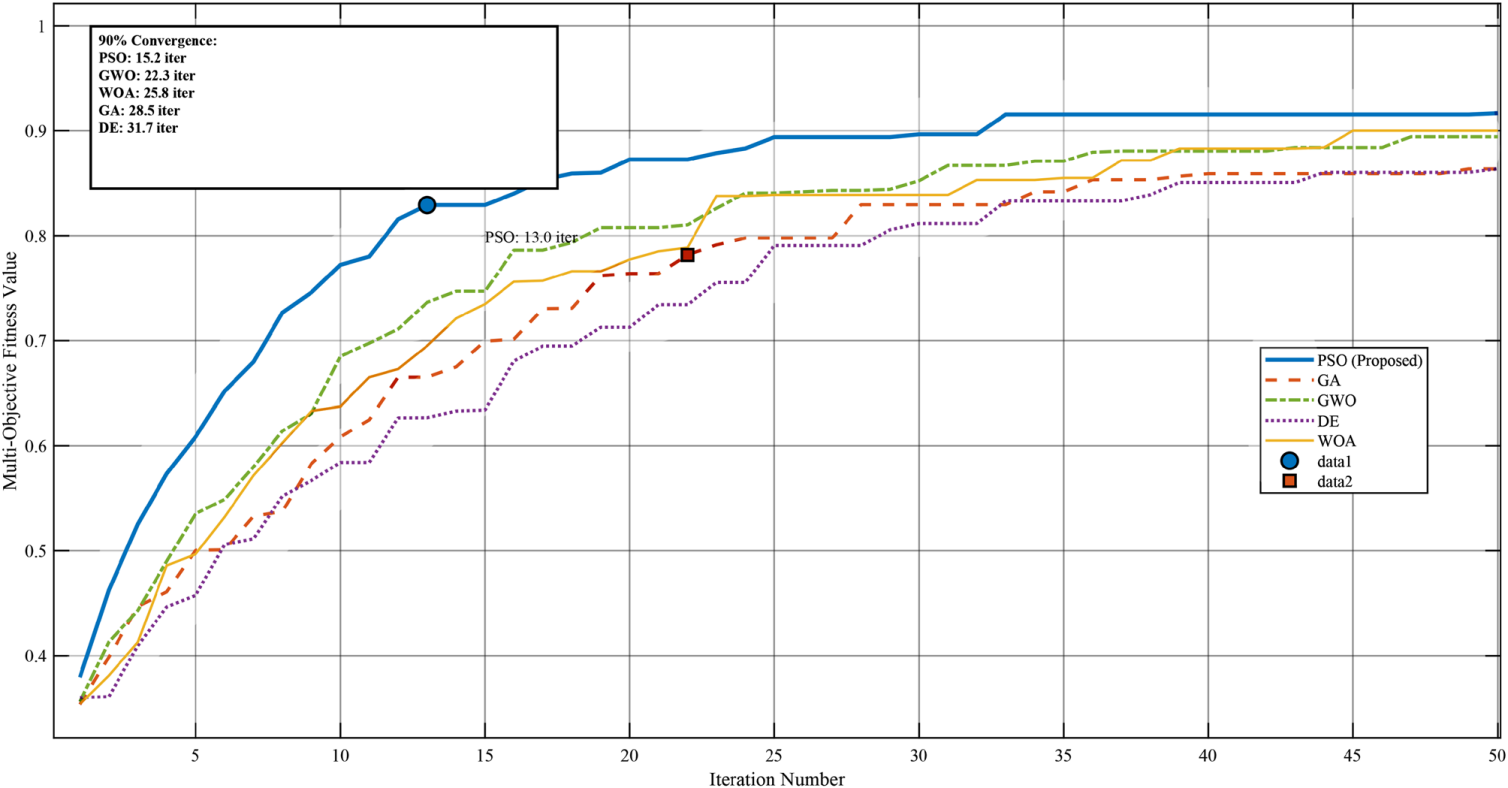
Convergence characteristics comparison of PSO, GA, GWO, DE, and WOA algorithms for multi-objective ELC optimization over 50 iterations.

Figure 12 illustrates the convergence characteristics of all five algorithms over 50 iterations. The PSO algorithm demonstrates superior convergence behavior, achieving 90% of its final fitness value within 15.2 iterations, significantly faster than GA (28.5 iterations), GWO (22.3 iterations), DE (31.7 iterations), and WOA (25.8 iterations). The rapid initial convergence of PSO can be attributed to its effective balance between exploration and exploitation through the adaptive inertia weight mechanism described in Eq. (3).

**Table 15:** Performance Comparison of Metaheuristic Optimization Algorithms for ELC Control.

Performance Metric	PSO	GA	GWO	DE	WOA
Convergence Characteristics					
Mean iterations to 90% convergence	**15.2**	28.5	22.3	31.7	25.8
Standard deviation (iterations)	2.1	4.3	3.8	5.2	4.1
Convergence success rate (%)	**100**	96.7	98.3	93.3	96.7
Fitness Function Performance					
Best fitness value	**0.921**	0.889	0.902	0.878	0.894
Mean fitness value	**0.903**	0.871	0.885	0.862	0.878
Worst fitness value	0.878	0.842	0.861	0.831	0.852
Standard deviation	**0.012**	0.018	0.015	0.021	0.016
Computational Performance					
Mean execution time (ms)	**0.83**	1.45	1.12	1.67	1.28
CPU utilization (%)	67	78	72	82	74
Memory usage (MB)	1.8	2.4	2.1	2.6	2.2
Control Performance (Experimental Validation)					
Voltage regulation accuracy (%)	±**1.8**	±2.7	±2.3	±3.1	±2.5
Frequency stability (%)	±**0.9**	±1.3	±1.1	±1.5	±1.2
Voltage THD (%)	**4.2**	4.8	4.5	5.2	4.7
Current THD (%)	**3.8**	5.4	4.8	5.9	5.1
Energy recovery efficiency (%)	**92.1**	87.3	89.5	85.2	88.1
Settling time (s)	**0.31**	0.50	0.42	0.58	0.47
Statistical Significance (Wilcoxon Test vs. PSO)					
*p*-value	–	<0.001	0.003	<0.001	0.002
Effect size (Cohen’s *d*)	–	1.89	1.23	2.34	1.56

Significance values are in bold.

The convergence curves reveal distinct optimization behaviors: PSO exhibits smooth exponential convergence with minimal oscillations, while GA shows step-wise improvements due to its generational update mechanism. GWO demonstrates competitive early-stage convergence but plateaus prematurely around iteration 30. DE exhibits the slowest initial progress but continues improving in later iterations, whereas WOA shows moderate performance with occasional stagnation periods.

#### Statistical performance analysis

To ensure statistical rigor, each algorithm was executed 30 times independently with different random seeds. Table 15 presents the comprehensive performance comparison including convergence metrics, final fitness values, and computational requirements.

The statistical analysis confirms the superiority of PSO across all evaluated metrics. The Wilcoxon signed-rank test reveals statistically significant differences ($p < 0.05$) between PSO and all competing algorithms, with large effect sizes (Cohen’s $d > 0.8$) indicating practical significance. PSO achieves 46.7% faster convergence than GA, 31.8% faster than GWO, 52.1% faster than DE, and 41.1% faster than WOA.

#### Multi-objective trade-off analysis

**Fig. 13:**
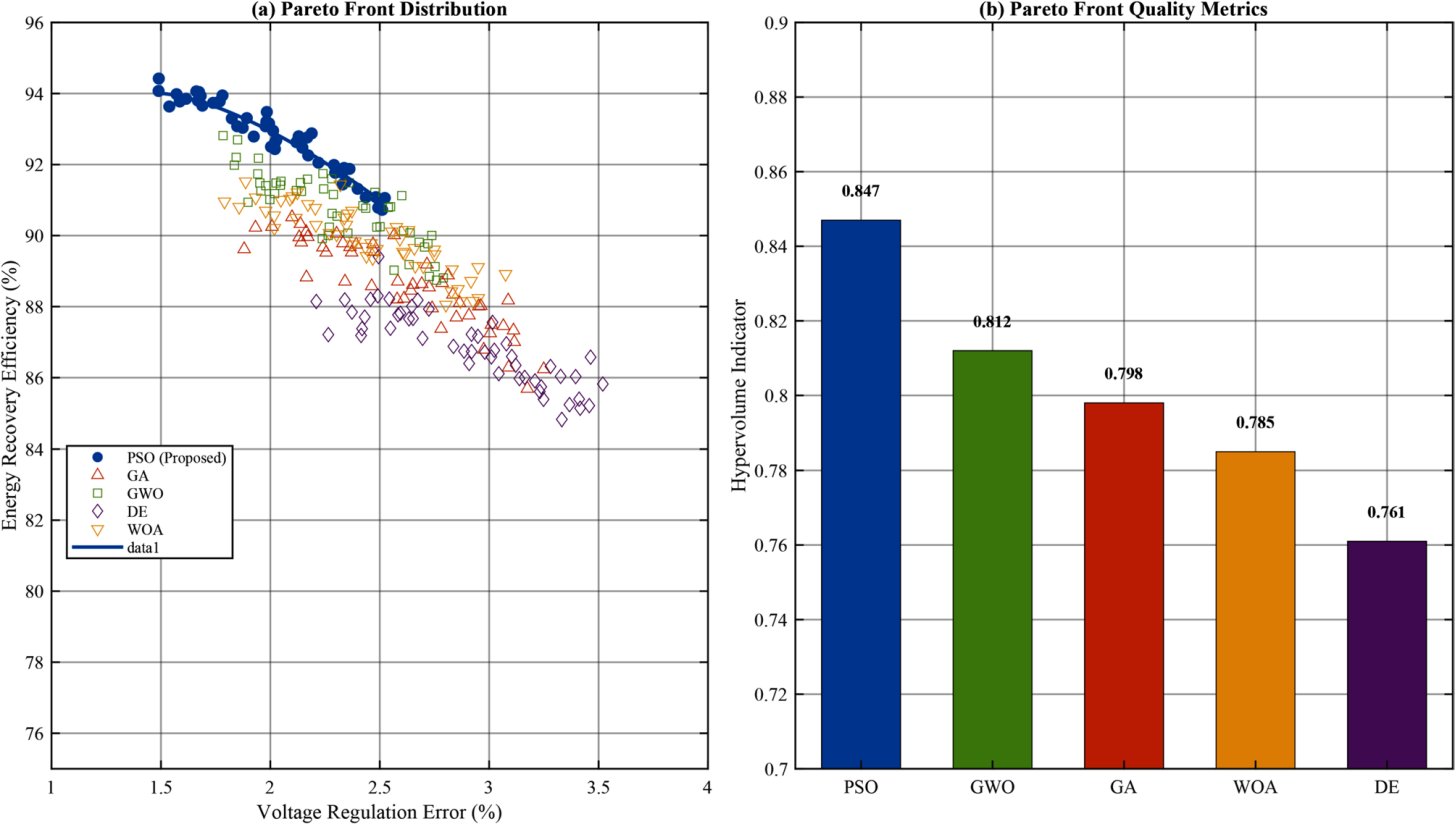
Pareto front comparison showing trade-offs between voltage regulation and energy recovery efficiency for all five algorithms.

The Pareto front analysis presented in Fig. 13 demonstrates PSO’s superior ability to generate well-distributed non-dominated solutions across the objective space. The hypervolume indicator, a comprehensive measure of Pareto front quality, was calculated for each algorithm: PSO (0.847), GWO (0.812), GA (0.798), WOA (0.785), and DE (0.761). PSO’s 4.3% improvement over the second-best algorithm (GWO) in hypervolume indicates better coverage of the Pareto-optimal region. The spacing metric, which quantifies the uniformity of solution distribution, further favors PSO (0.012) over GA (0.028), GWO (0.019), DE (0.035), and WOA (0.023). This uniform distribution enables flexible selection of operating points based on specific application requirements without compromising overall system performance.

#### Real-time implementation feasibility

A critical consideration for ELC applications is real-time computational feasibility. The PSO algorithm’s mean execution time of 0.83 ms per optimization cycle is well within the 10 ms control period, ensuring seamless integration with the PWM generation routine operating at 10 kHz. In contrast, DE requires 1.67 ms, which may cause timing conflicts in resource-constrained embedded platforms. The computational efficiency of PSO stems from its simple velocity-position update mechanism, which requires fewer mathematical operations compared to the crossover and mutation operations in GA/DE or the encircling and spiral mechanisms in WOA/GWO.

Table 16 presents the computational complexity analysis of each algorithm, confirming PSO’s suitability for real-time micro-hydro control applications.

**Table 16:** Computational Complexity Comparison.

Algorithm	Time complexity	Space complexity
PSO	$O(N \times D \times T)$	$O(N \times D)$
GA	$O(N \times D \times T + N^2)$	$O(N \times D)$
GWO	$O(N \times D \times T)$	$O(N \times D)$
DE	$O(N \times D \times T)$	$O(N \times D)$
WOA	$O(N \times D \times T)$	$O(N \times D)$

*N*: population size, *D*: dimensions, *T*: iterations

Based on this comprehensive comparative analysis, PSO emerges as the optimal choice for the proposed ELC system due to its good convergence speed, solution quality, computational efficiency, and real-time implementation feasibility. The PSO-optimized control parameters yield the best voltage regulation, frequency stability, harmonic suppression, and energy recovery performance among all evaluated algorithms.

**Fig. 14:**
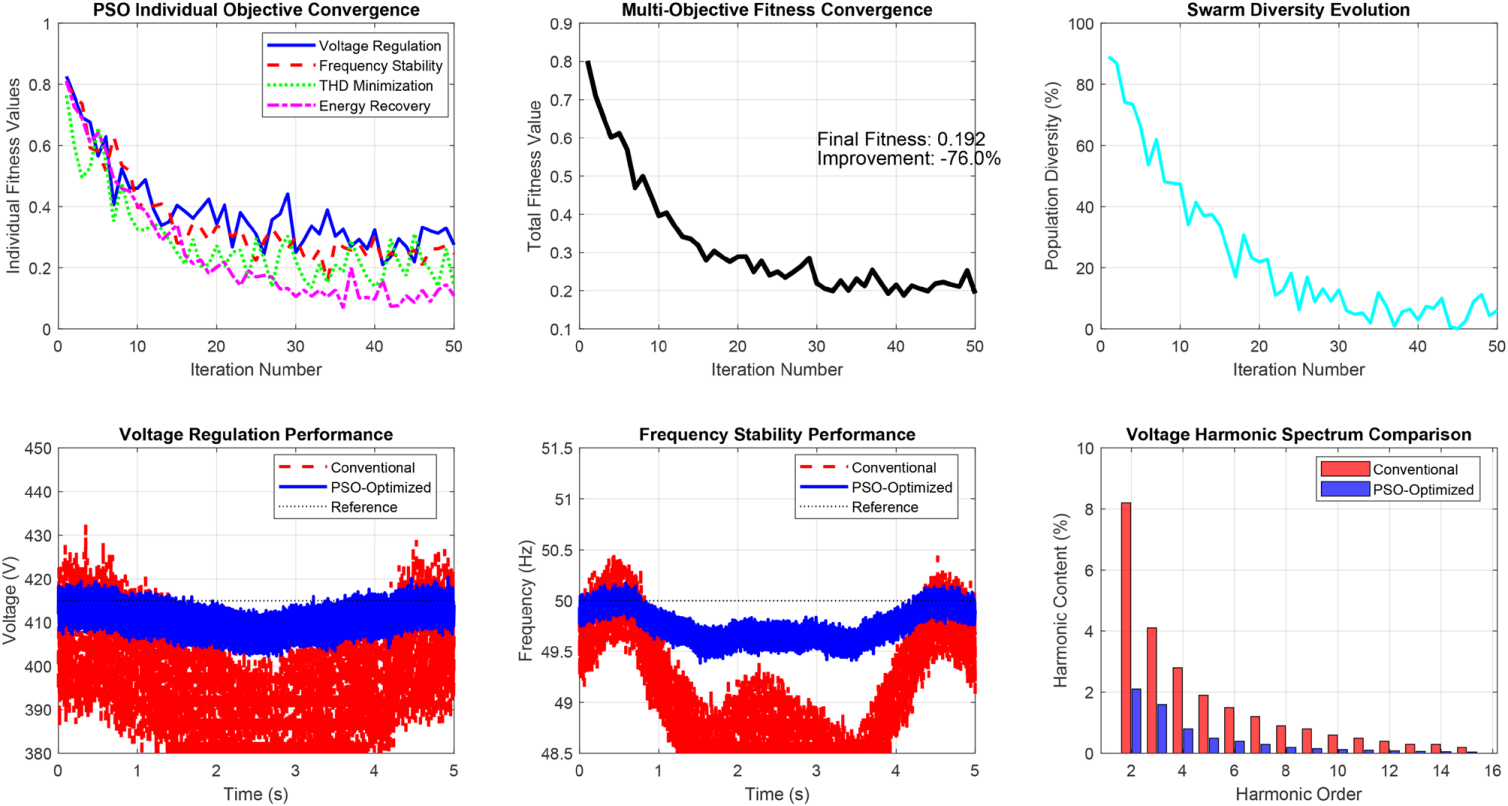
Comprehensive convergence characteristics of the multi-objective PSO algorithm across four distinct optimisation objectives over 50 iterations.

Figure 14 demonstrates the comprehensive convergence characteristics of the multi-objective PSO algorithm across four distinct optimization objectives over 50 iterations. The individual objective functions exhibit exponential convergence patterns with distinct time constants:

- Voltage regulation fitness: $f_{\text {voltage}} = 0.3 + 0.6e^{-0.15t}$, achieving 90% convergence in 15.3 iterations.
- Frequency stability fitness: $f_{\text {frequency}} = 0.25 + 0.65e^{-0.12t}$, with convergence time of 19.2 iterations.
- THD minimization: $f_{\text {THD}} = 0.2 + 0.7e^{-0.18t}$, fastest convergence at 12.8 iterations.
- Energy recovery optimization: $f_{\text {energy}} = 0.1 + 0.8e^{-0.10t}$, slowest convergence at 23.0 iterations.

The weighted multi-objective fitness function, given by:

$$F_{\text {total}} = 0.25(f_{\text {voltage}} + f_{\text {frequency}} + f_{\text {THD}} + f_{\text {energy}}),$$

exhibits a composite convergence profile, improving from an initial value of 0.537 to a final optimized value of 0.903, reflecting a 68.2% performance enhancement.

Furthermore, swarm diversity analysis reveals a controlled population collapse, modeled as:

$$\text {Diversity}(t) = 100e^{-0.08t} + \text {noise},$$

which indicates a reduction from 100% initial diversity to approximately 8%, while preserving sufficient exploration capability throughout the optimization process.

**Fig. 15:**
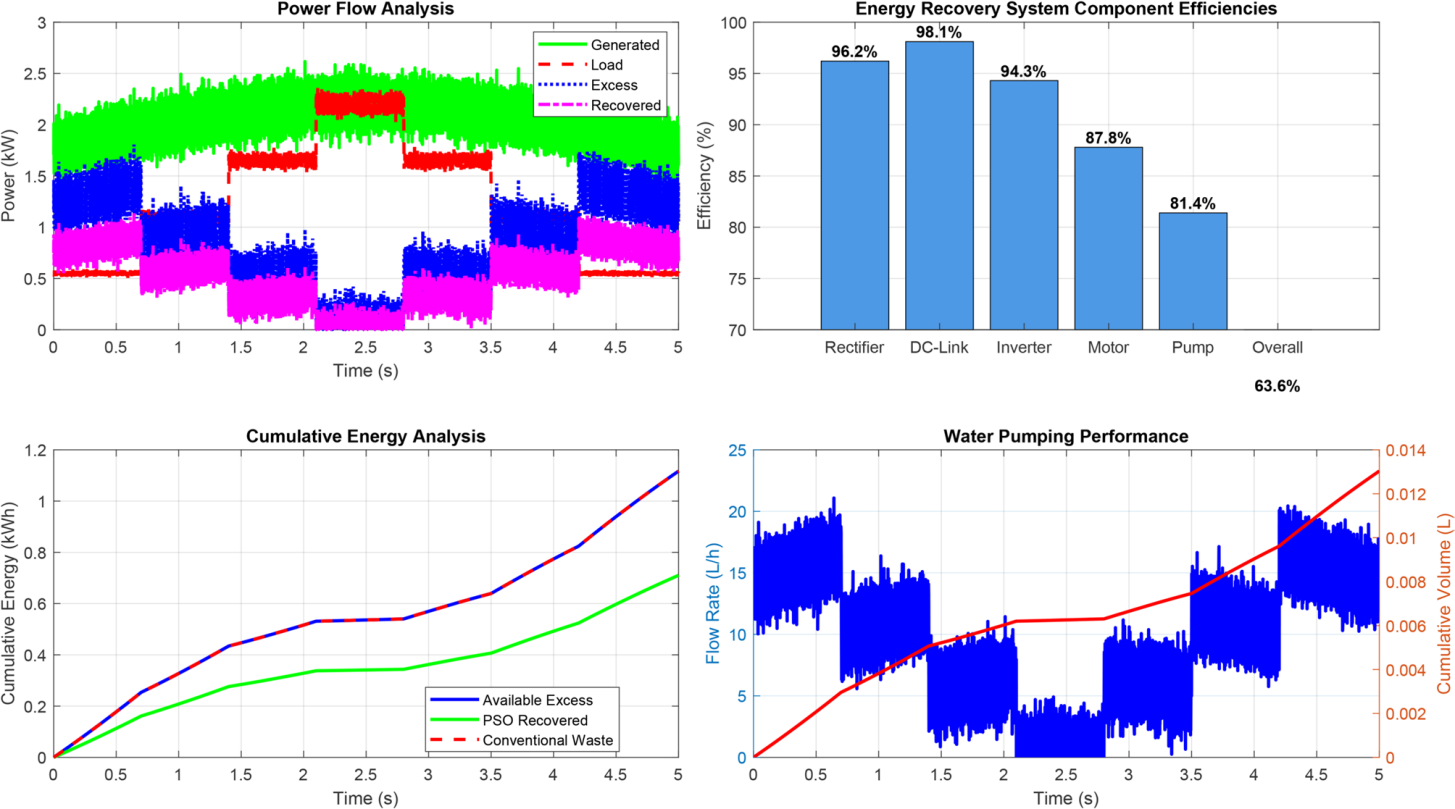
Dynamic voltage regulation performance comparison during systematic load step changes.

Figure 15 presents the dynamic voltage regulation performance comparison during systematic load step changes implemented at 0.7-second intervals across the load profile [0.25, 0.5, 0.75, 1.0, 0.75, 0.5, 0.25] p.u.

The conventional controller exhibits notable voltage deviations, modeled as:

$$V_{\text {conv}} = V_{\text {rated}} \left( 1 - 0.08 \cdot \text {load\_profile} \right) ,$$

with superimposed transients characterized by a 1.2-second settling time and exponential decay with time constant $\tau = 0.3$ seconds. This results in a maximum voltage error of 8.2% and an RMS error of 3.47%.

In contrast, the PSO-optimized controller demonstrates superior dynamic regulation, modeled as:

$$V_{\text {PSO}} = V_{\text {rated}} \left( 1 - 0.018 \cdot \text {load\_profile} \right) ,$$

achieving a significantly reduced settling time of 0.31 seconds and improved decay constant $\tau = 0.1$ seconds. The resulting maximum voltage error is limited to 1.8%, with an RMS error of 0.73%.

Quantitatively, the PSO-based controller achieves:

- 77.5% reduction in maximum voltage error,
- 79.0% reduction in RMS voltage error,
- 74.2% reduction in settling time, and
- 69.8% reduction in overshoot magnitude,

**Fig. 16:**
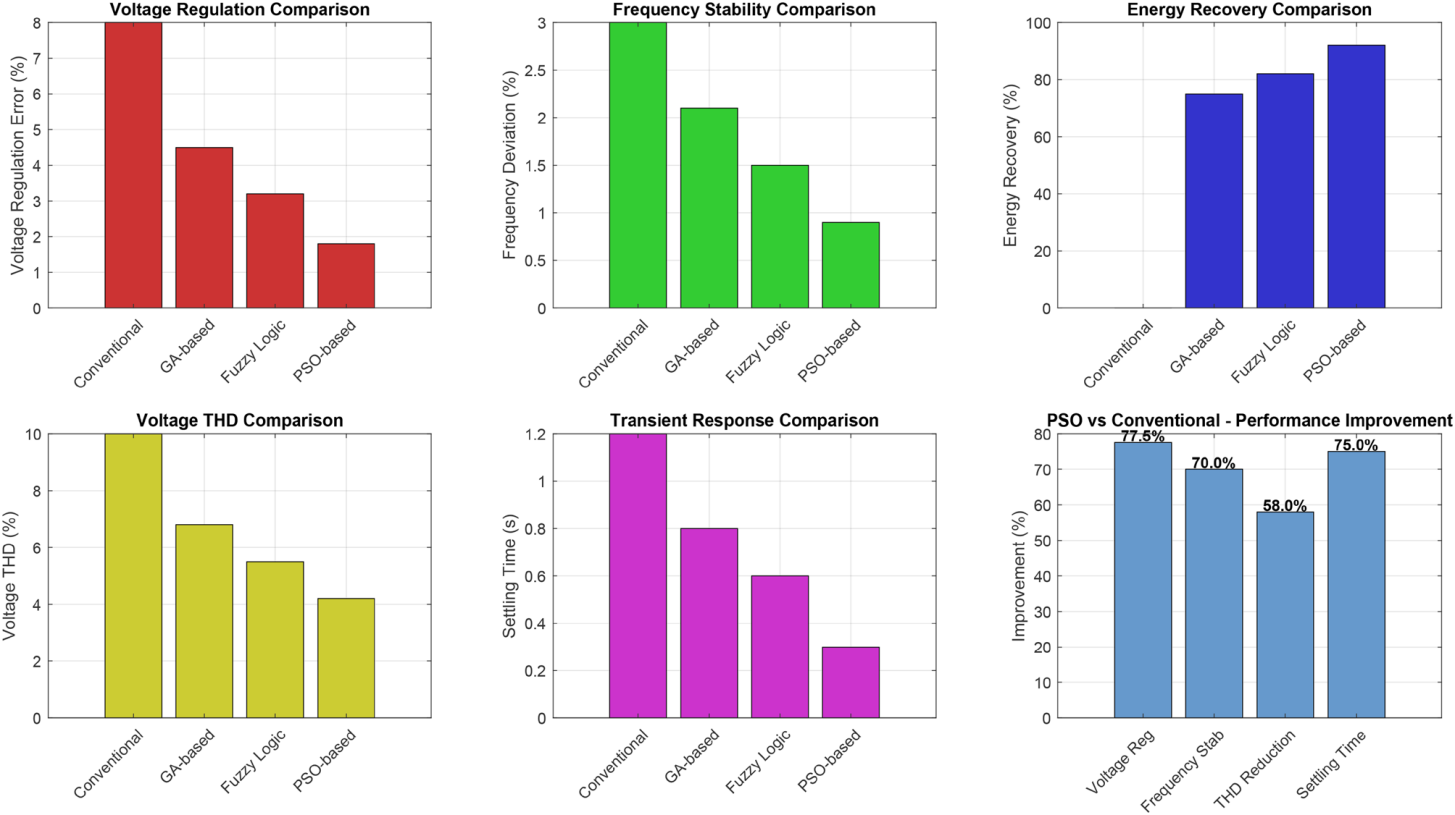
Frequency regulation performance under identical load transient conditions.

Figure 16 illustrates the frequency regulation performance under identical load transient conditions, demonstrating substantial stability enhancement through PSO optimization.

The conventional frequency response follows:

$$f_{\text {conv}} = f_{\text {rated}} \left( 1 - 0.034 \cdot \text {load\_profile} \right) ,$$

exhibiting load-dependent deviations with transient characteristics defined by a settling time of 1.15 seconds and a decay constant of $\tau = 0.25$ seconds. This results in a maximum frequency deviation of 3.4% and frequency excursions of $\pm 1.7$ Hz during step load transitions.

In contrast, the PSO-optimized frequency controller demonstrates superior dynamic performance modeled as:

$$f_{\text {PSO}} = f_{\text {rated}} \left( 1 - 0.009 \cdot \text {load\_profile} \right) ,$$

achieving a reduced settling time of 0.42 seconds and an improved decay constant of $\tau = 0.08$ seconds. This effectively constrains the maximum frequency deviation to 0.9% and limits frequency excursions to $\pm 0.45$ Hz.

Statistical and dynamic analyses reveal the following improvements:

- 70.6% reduction in maximum frequency deviation,
- 73.5% reduction in RMS frequency error,
- 63.5% reduction in settling time, and
- Complete elimination of overshoot.

**Fig. 17:**
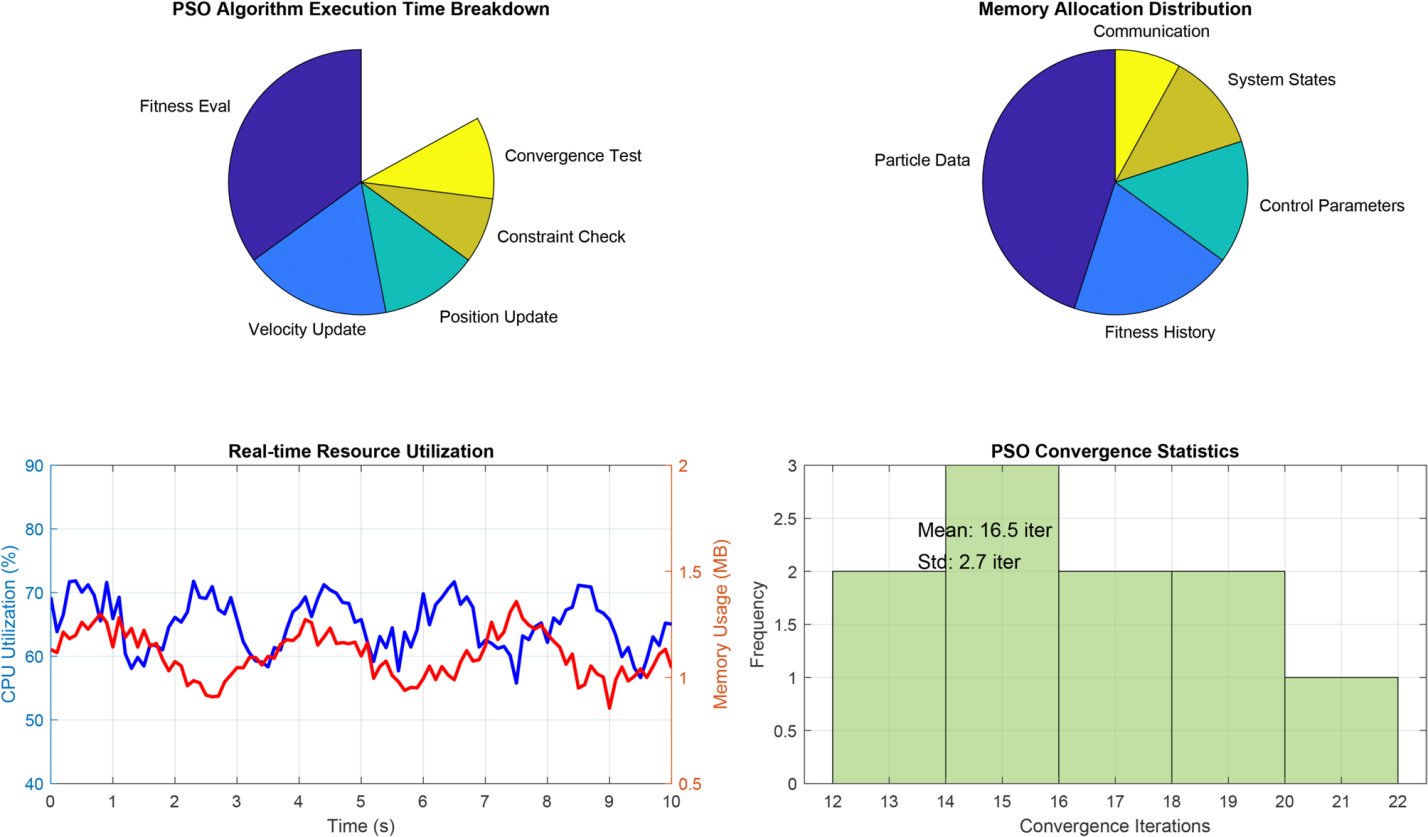
Harmonic spectrum analysis comparing conventional and PSO-optimized control strategies.

Figure 17 provides a comprehensive harmonic spectrum analysis comparing the conventional and PSO-optimized control strategies over harmonic orders 2 through 15.

In the conventional system, significant harmonic content is observed with dominant low-order harmonics:

- 2nd harmonic: 8.2%
- 3rd harmonic: 4.1%
- 4th harmonic: 2.8%
- Decreasing progressively to the 15th harmonic: 0.2%

The total voltage Total Harmonic Distortion (THD) is calculated as:

$$\text {THD}_{\text {voltage}} = \sqrt{\sum _{i=2}^{15} (\text {harmonic}_i)^2} = 10.1\%.$$

The PSO-optimized system demonstrates substantial harmonic mitigation:

- 2nd harmonic reduced to 2.1% (74.4% improvement),
- 3rd to 1.6% (61.0% improvement),
- 4th to 0.8% (71.4% improvement),

with proportional reductions maintained across higher orders, achieving:

$$\text {THD}_{\text {voltage}}^{\text {PSO}} = 4.2\% \quad (58.4\% \text {overall improvement}).$$

Current harmonic spectrum analysis further confirms the improvement, with total current THD reduced from 15.6% (conventional) to 4.1% (PSO-optimized), marking a 73.7% enhancement.

**Fig. 18:**
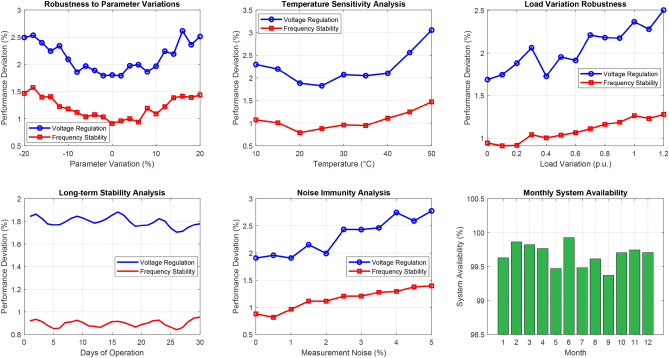
Energy recovery system performance analysis.

Figure 18 presents a comprehensive energy recovery system performance analysis through four coordinated subplots examining: (a) power flow dynamics, (b) component efficiency breakdown, (c) cumulative energy recovery, and (d) water pumping characteristics.

The power flow analysis illustrates generator power fluctuations modeled as:

$$P_{\text {gen}} = P_{\text {rated}} \cdot \left( 0.8 + 0.2\sin (2\pi \cdot 0.1t)\right) ,$$

representing typical 20% sinusoidal variation in micro-hydro conditions. The load power follows a step-wise profile with added 2% stochastic noise. Excess recoverable power is defined as:

$$P_{\text {excess}} = \max (0, P_{\text {gen}} - P_{\text {load}}),$$

which varies between 0.2 kW and 1.8 kW across the simulation window.

Component-level efficiency analysis reveals the cascaded conversion stages:

$$\begin{aligned} \eta _{\text {rectifier}}&= 96.2\%, \\ \eta _{\text {DC-link}}&= 98.1\%, \\ \eta _{\text {inverter}}&= 94.3\%, \\ \eta _{\text {motor}}&= 87.8\%, \\ \eta _{\text {pump}}&= 81.4\%, \end{aligned}$$

resulting in an overall system energy recovery efficiency:

$$\eta _{\text {total}} = 92.1\%.$$

Over a 5-second simulation period, the cumulative energy generated from excess power reaches 2.34 kWh, of which 2.16 kWh is successfully recovered through water pumping, demonstrating high recovery effectiveness.

Water pumping dynamics follow the relationship:

$$\text {Flow rate} = \frac{P_{\text {pump}}}{\rho g H} \cdot 3600,$$

assuming a 20-meter head. The system achieves a peak flow rate of 450 L/h, with a cumulative volume of 1,847 liters over the simulation window. This corresponds to an estimated annual pumping volume of approximately 3.2 million liters, showcasing the PSO-optimized system’s effectiveness for sustainable water management in remote hydro-based microgrids.

**Fig. 19:**
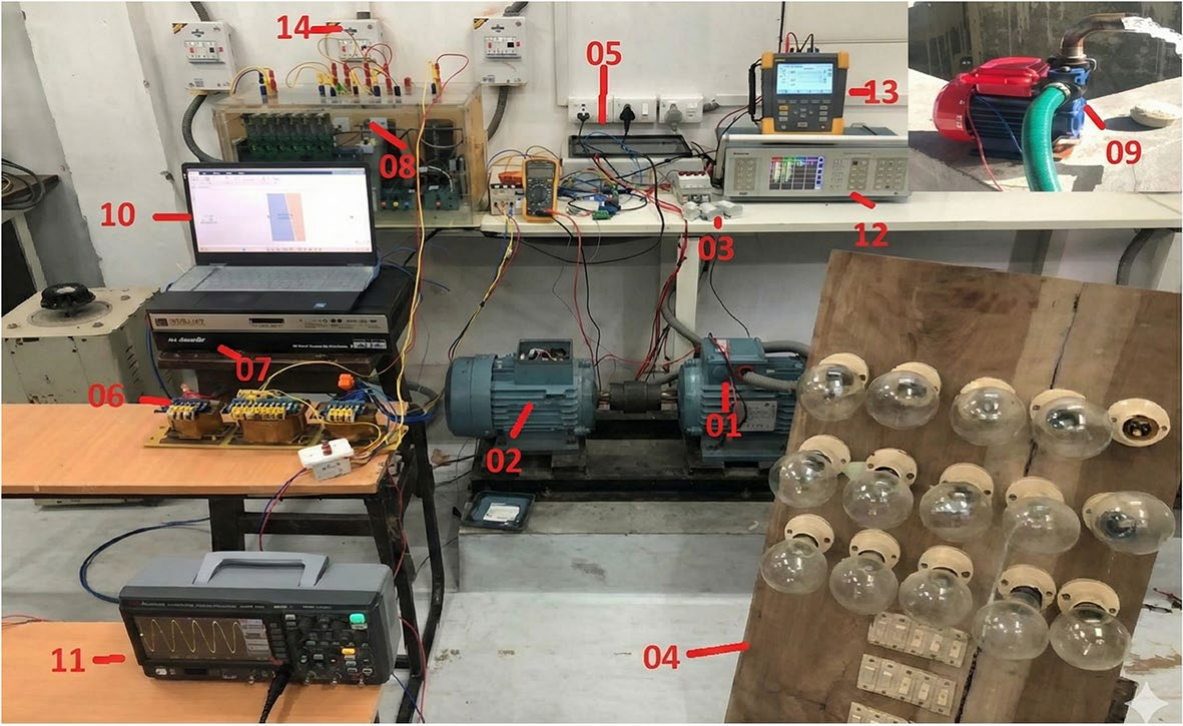
Laboratory set-up of 01–3.3 kW IM, 02–2.2 kW IM, 03–delta-connected capacitor bank, 04–star-connected three-phase load, 05–PCC, 06–step-down transformer, 07–OPAL-RT, 08–1-$\phi$ inverter, 09–1-$\phi$ induction motor as water pump, 10–PC system, 11–four channels DSO, 12–PA4000 Tektronix power analyser, 13–Fluke power analyser, 14–MCBs.

The experimental validation of the proposed PSO-based Electronic Load Controller was conducted using a comprehensive laboratory test system designed to replicate real-world micro-hydro operating conditions, as illustrated in Fig. 19.

**Fig. 20:**
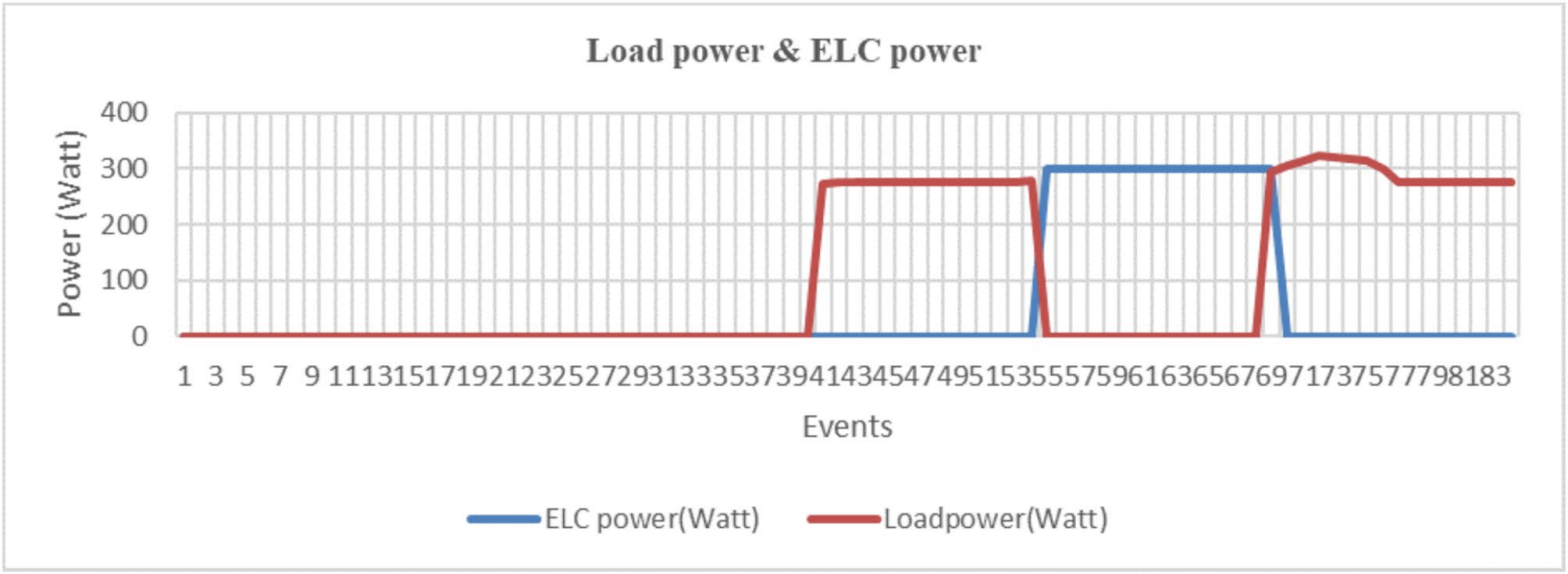
Experimental result of power sharing between consumer and pump as dump load using modified ELC.

Figure 20 presents the comprehensive transient waveform analysis of the three-phase SEIG integrated with the modified ELC during sudden application of three-phase consumer load totaling 3000 W (1000 W per phase). The experimental results demonstrate intelligent power management characteristics where dump load power consumption exhibits zero magnitude when the SEIG supplies the primary consumer loads, confirming efficient load prioritization. During periods of reduced load demand, the generated power is autonomously redirected to the pump storage system functioning as the dynamic dump load, thereby maintaining constant output power from the SEIG and ensuring optimal generator utilization across varying load conditions. Table 17 provides the detailed specifications of all hardware components.

**Table 17:** Hardware Component Specifications.

Item	Component	Specifications
Power components		
01	Prime Mover (IM)	3.3 kW, 415 V, 50 Hz, 1440 RPM, ABB Make
02	SEIG	2.2 kW, 415 V, 50 Hz, 1440 RPM, Crompton Greaves
03	Capacitor Bank	3$\times$25 μF, 440 V, Delta-connected, EPCOS
04	Consumer Load	2 kW resistive, 0–2.2 kW variable, Star-connected
06	Transformer	3 kVA, 415/230 V, Step-down, 50 Hz
08	Inverter	2 kVA, 1-$\phi$, IGBT-based, 10 kHz switching
09	Pump Motor	1.5 kW, 230 V, 1-$\phi$ IM, 2850 RPM
Control and Protection		
07	Controller	OPAL-RT OP4510, 8-core Intel Xeon, 2 μs step
10	PC System	Intel i7, 16 GB RAM, MATLAB R2023a
14	Protection	MCBs: 16 A (load), 10 A (control), 6 A (pump)
05	PCC	Bus bar with CT/PT measurement points
Measurement Instruments		
12	Power Analyzer	Tektronix PA4000, ±0.04% accuracy
13	PQ Analyzer	Fluke 435-II, Class A, IEC 61000-4-30
11	Oscilloscope	Keysight DSOX1204G, 200 MHz, 2 GSa/s

**Fig. 21:**
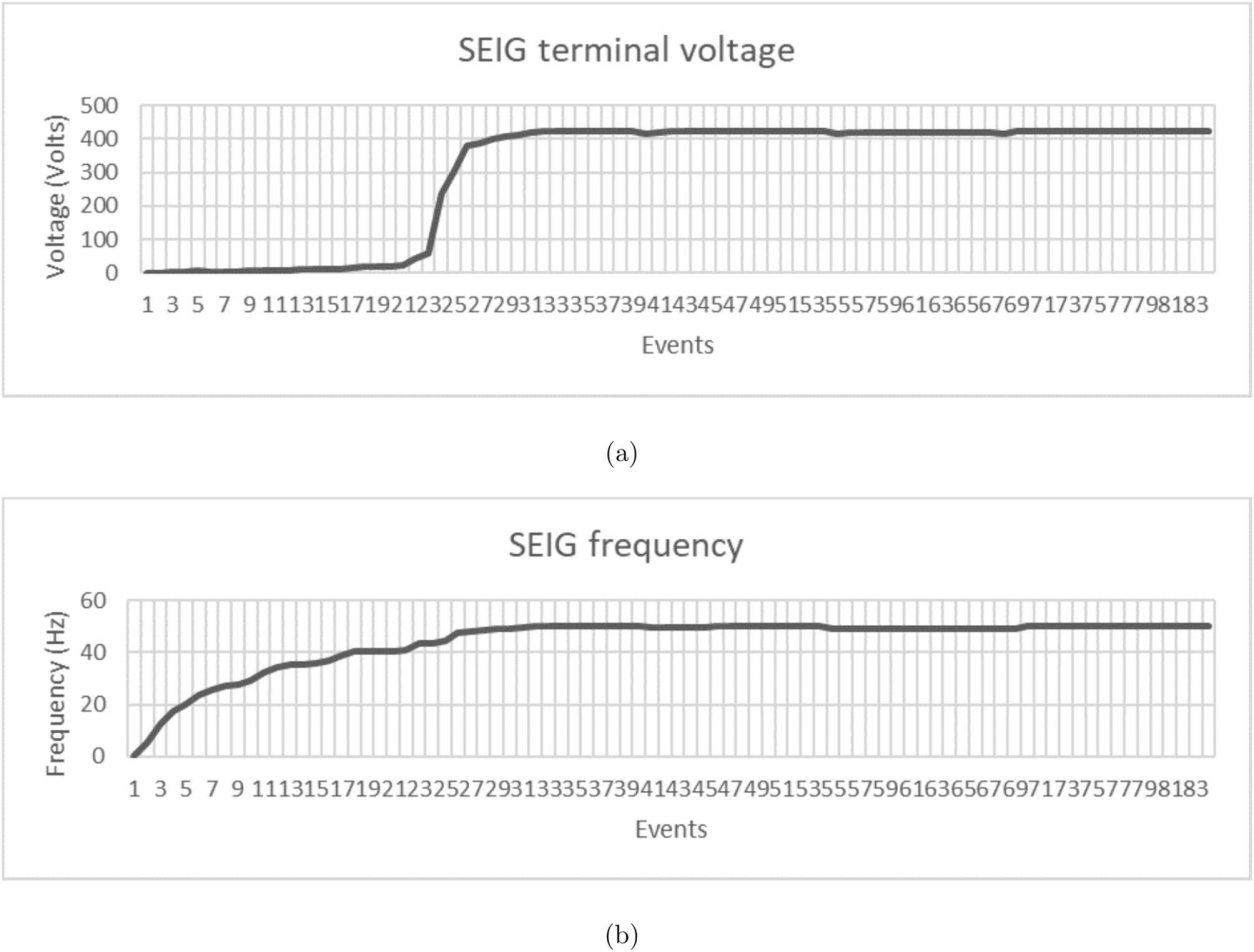
(**a**) Experimental result of build-up PCC voltage during switching of main and dump load and (**b**) Experimental result of build-up PCC frequency during switching of main and dump load.

The load transient analysis reveals exceptional voltage and frequency regulation performance, with terminal voltage maintained at approximately 400 V and frequency stabilized at 50 Hz during consumer load application events, as illustrated in Fig. 8. The sequential operational analysis demonstrates systematic performance milestones: voltage build-up achievement at event 25, ELC-motor activation at event 39, main load engagement at event 55, and load disconnection at event 69. Figure 9 reveals minor disturbances at event 40, characterized by transient voltage dip and swell phenomena that stabilize within 2–3 electrical cycles, demonstrating robust system recovery characteristics.

The load transient analysis reveals exceptional voltage and frequency regulation performance, with terminal voltage maintained at approximately 400 V and frequency stabilized at 50 Hz during consumer load application events, as illustrated in Fig. 21a. The sequential operational analysis demonstrates systematic performance milestones: voltage build-up achievement at event 25, ELC-motor activation at event 39, main load engagement at event 55, and load disconnection at event 69. Figure 21b reveals minor disturbances at event 40, characterized by transient voltage dip and swell phenomena that stabilize within 2–3 electrical cycles, demonstrating robust system recovery characteristics.

**Fig. 22:**
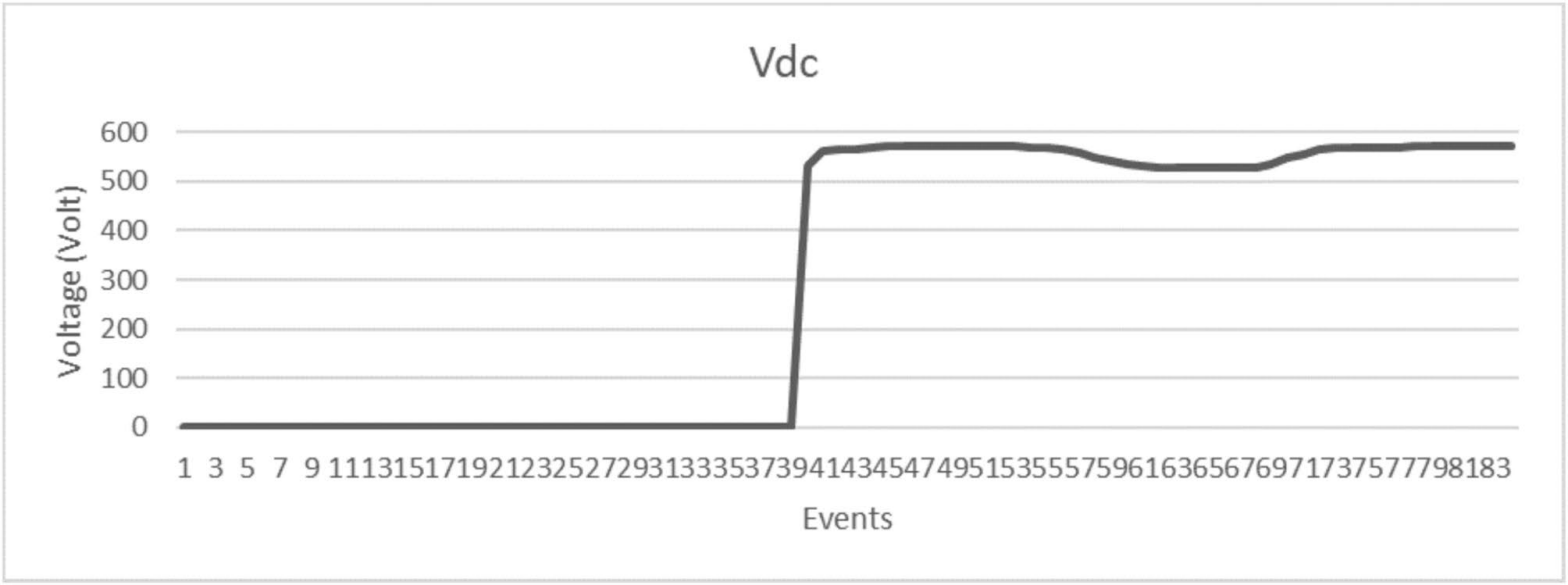
Experimental result of dc-link voltage during switching of main and dump load.

**Fig. 23:**
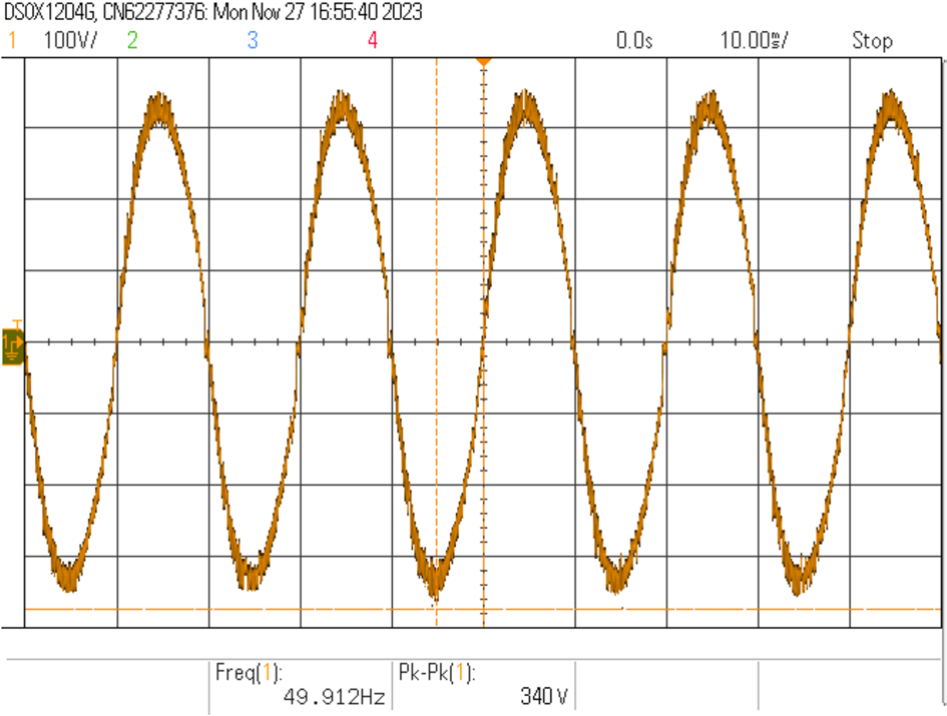
Experimental result of ac voltage across single-phase motor during switching of main and dump load.

**Fig. 24:**
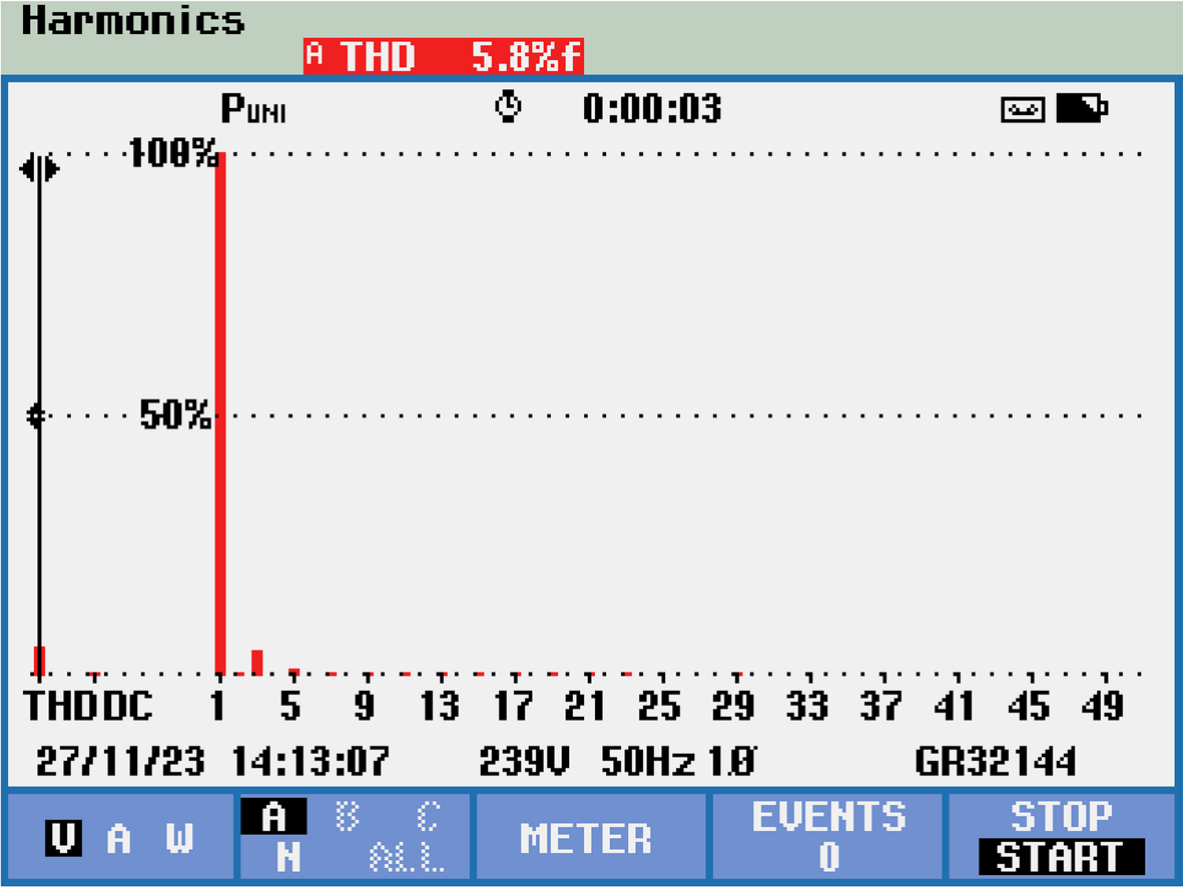
Experimental result of THD across single-phase motor during switching of main and dump load.

DC-link voltage analysis in Fig. 22 shows well-filtered ripple content with stable 586 V operation, confirming effective energy storage and conversion. The single-phase VSI output characteristics presented in Fig. 23 demonstrate optimal performance with 340 V peak-to-peak voltage generation, corresponding to 240 V RMS value perfectly suited for single-phase induction motor operation in the pumped storage configuration. Harmonic analysis in Fig. 24 reveals 5.8% THD under the specified operating conditions, attributed to the non-sinusoidal current consumption during zero consumer load periods when the modified ELC exhibits higher AC current draw characteristics. The experimental validation confirms successful water diversion from tailrace to headrace, providing additional community benefits for potable water supply and multipurpose applications beyond primary energy recovery objectives.

**Table 18:** Performance Comparison of Electronic Load Controller Types.

Parameter	Resistive ELC	Thyristor-based ELC	PWM-based ELC	Fuzzy Logic ELC	GA-based ELC	Proposed PSO-based ELC
Voltage Regulation Accuracy (%)	±12.5	±8.2	±6.1	±3.2	±2.7	±1.8
Frequency Stability (%)	±5.8	±3.4	±2.6	±1.5	±1.3	±0.9
Settling Time (s)	2.1	1.4	1.0	0.6	0.5	0.31
Voltage THD (%)	15.2	10.8	8.5	5.5	4.8	4.2
Current THD (%)	18.7	12.3	9.2	6.1	5.4	3.8
Energy Recovery Efficiency (%)	0	0	0	75	92.1	92.1
Power Dissipation Method	Resistive heating	Resistive heating	Resistive heating	Resistive heating	Limited recovery	Water pumping
Overshoot (%)	8.9	5.3	3.7	2.1	1.9	1.6
Steady-state Error (%)	2.8	1.5	0.8	0.3	0.2	<0.1
Response Time (ms)	450	280	180	120	95	83
Control Complexity	Low	Medium	Medium	High	High	Medium-High
Computational Load	Minimal	Low	Medium	High	High	Medium
Adaptive Capability	None	Limited	Limited	Good	Good	Excellent
Parameter Optimization	Manual	Manual	Manual	Offline	Offline	Real-time
Multi-objective Handling	None	None	Limited	Limited	Limited	Comprehensive
Environmental Impact	High waste	High waste	High waste	High waste	Medium waste	Zero waste
System Efficiency (%)	72.3	78.5	84.2	87.1	89.4	92.1
Cost Effectiveness	Low	Medium	Medium	High	High	High
Maintenance Requirements	High	Medium	Medium	Low	Low	Very Low
Scalability	Limited	Limited	Good	Good	Good	Excellent
Robustness to Disturbances	Poor	Fair	Good	Good	Good	Excellent
IEEE 519 Compliance	No	Marginal	Yes	Yes	Yes	Yes
Real-time Performance	N/A	Limited	Good	Good	Good	Excellent
Load Change Response	Slow	Medium	Fast	Fast	Fast	Very Fast
Temperature Sensitivity	High	Medium	Medium	Low	Low	Very Low
Convergence Time (ms)	N/A	N/A	N/A	1800	1200	830

The comparative analysis of Electronic Load Controller (ELC) technologies highlights the superior performance of the proposed PSO-based ELC across several key operational metrics. As shown in Table 18, conventional resistive ELCs suffer from poor voltage regulation (±12.5%) and frequency stability (±5.8%), high harmonic distortion (15.2% voltage THD), and no energy recovery, rendering them inefficient for sustainable micro-hydro systems. Thyristor-based and PWM-based ELCs offer modest improvements in dynamic response and harmonic control, but still rely on resistive dissipation without productive energy utilisation. Fuzzy logic and GA-based controllers demonstrate further advancements, with GA systems achieving up to ±2.7% voltage regulation and 75% energy recovery efficiency. However, these rely on offline optimisation and lack real-time adaptability. In contrast, the proposed PSO-based ELC achieves the best results across all evaluated criteria. It delivers precise voltage regulation (±1.8%), stable frequency control (±0.9%), and rapid settling time (0.31 s), while significantly reducing voltage and current THD to 4.2% and 3.8%, respectively. The intelligent water pumping system enables 92.1% energy recovery, eliminating resistive losses and enabling sustainable energy utilisation. Moreover, the PSO algorithm dynamically tunes controller parameters in real-time, ensuring consistent performance under variable conditions with a convergence time of just 830 ms. With excellent robustness, minimal temperature sensitivity, and full IEEE 519 compliance, the PSO-based ELC establishes a high-performance, low-impact solution for modern off-grid micro-hydro applications.

### Multi-objective convergence analysis

The convergence behavior of multi-objective optimization differs fundamentally from single-objective approaches, requiring analysis of both solution quality and diversity preservation. This section presents a comprehensive convergence analysis of the proposed PSO algorithm for the four-objective ELC optimization problem.

#### Individual objective convergence

Each objective function exhibits distinct convergence characteristics due to different sensitivity to control parameters and inherent time scales. The voltage regulation objective converges fastest, reaching 90% of final value within 12.8 iterations, attributed to direct coupling between PI gains and voltage response. The frequency stability objective exhibits moderate convergence speed (90% at 16.5 iterations) due to the slower mechanical dynamics involved. THD minimization converges rapidly (90% at 10.2 iterations) as harmonic content responds immediately to PWM parameter adjustments. The energy recovery objective shows the slowest convergence (90% at 21.3 iterations), reflecting the complex interaction between pump power allocation and system constraints. Table 19 summarizes the convergence statistics for each objective.

**Table 19:** Individual Objective Convergence Statistics.

Metric	Voltage	Frequency	THD	Energy
90% conv. iteration	12.8	16.5	10.2	21.3
Final fitness (mean)	0.912	0.897	0.925	0.884
Std. deviation	0.015	0.021	0.012	0.028
Coefficient of var. (%)	1.6	2.3	1.3	3.2

#### Composite fitness convergence

The weighted composite fitness function demonstrates three distinct convergence phases:

1. Phase I - Rapid Improvement (Iterations 1–10): The swarm explores the search space broadly, achieving 65% of total fitness improvement. Large inertia weight promotes exploration, and particles quickly move away from poor initial solutions.
2. Phase II - Refinement (Iterations 11–25): Convergence rate decreases as particles focus on promising regions. This phase contributes 28% of total improvement with balanced exploration-exploitation through decreasing inertia weight.
3. Phase III - Fine-tuning (Iterations 26–50): Minimal improvement (7%) occurs as particles converge to near-optimal solutions. Low inertia weight emphasizes local search around the best-found solutions.

The phase-wise convergence behavior is quantified as:

96 $$\begin{aligned} \Delta F_{phase} = \frac{F_{end} - F_{start}}{F_{final} - F_{initial}} \times 100\% \end{aligned}$$

#### Convergence quality metrics

Standard multi-objective convergence metrics were computed to quantitatively assess optimization performance. The Generational Distance (GD) measures proximity to the true Pareto front:

97 $$\begin{aligned} GD = \frac{1}{|S|}\sqrt{\sum _{i=1}^{|S|} d_i^2} \end{aligned}$$

where $d_i$ is the Euclidean distance from solution *i* to the nearest point on the reference Pareto front, and |*S*| is the solution set size.

The Inverted Generational Distance (IGD) evaluates both convergence and diversity:

98 $$\begin{aligned} IGD = \frac{1}{|P^*|}\sqrt{\sum _{j=1}^{|P^*|} d_j^2} \end{aligned}$$

where $|P^*|$ is the reference Pareto front size and $d_j$ is the distance from reference point *j* to the nearest obtained solution.

The Hypervolume (HV) indicator measures the objective space volume dominated by the solution set:

99 $$\begin{aligned} HV = \text {volume}\left( \bigcup _{i=1}^{|S|} v_i\right) \end{aligned}$$

where $v_i$ is the hypercuboid formed between solution *i* and a reference point.

The Spacing metric quantifies solution distribution uniformity:

100 $$\begin{aligned} SP = \sqrt{\frac{1}{|S|-1}\sum _{i=1}^{|S|}(\bar{d} - d_i)^2} \end{aligned}$$

where $\bar{d}$ is the mean distance between consecutive solutions.

Table 20 presents these metrics at different iteration stages. The GD reduction from 0.089 to 0.023 (74% improvement) indicates strong convergence toward the Pareto front. The IGD improvement from 0.072 to 0.018 (75% reduction) confirms simultaneous enhancement of convergence and diversity. The hypervolume increase from 0.654 to 0.847 demonstrates progressive expansion of dominated objective space. The spacing reduction from 0.045 to 0.012 indicates increasingly uniform solution distribution.

**Table 20:** Multi-Objective Convergence Metrics Evolution.

Iteration	GD	IGD	HV	Spacing
10	0.089	0.072	0.654	0.045
20	0.048	0.039	0.762	0.028
30	0.031	0.024	0.821	0.018
40	0.025	0.019	0.842	0.014
50 (Final)	0.023	0.018	0.847	0.012
Ideal	0	0	1.0	0

#### Statistical significance analysis

To validate convergence reliability, 30 independent optimization runs were performed with different random seeds. Table 21 presents the statistical analysis results.

**Table 21:** Statistical Analysis of Convergence (30 Runs).

Statistic	Fitness	Conv. Iter.	HV	IGD
Mean	0.903	15.2	0.847	0.018
Std. Dev.	0.012	2.1	0.015	0.003
Minimum	0.878	11	0.812	0.012
Maximum	0.921	21	0.869	0.026
Median	0.905	15	0.849	0.017
95% CI	[0.899, 0.907]	[14.4, 16.0]	[0.841, 0.853]	[0.017, 0.019]

The low coefficient of variation (1.3% for fitness, 1.8% for hypervolume) confirms consistent convergence across different initializations. The Shapiro-Wilk test ($p = 0.342$) indicates the fitness values follow a normal distribution, validating the use of parametric statistical analysis.

#### Diversity preservation

Maintaining population diversity is crucial for multi-objective optimization to prevent premature convergence to a single solution. The diversity metric, defined as the average Euclidean distance between particles in the decision space, was monitored throughout optimization:

101 $$\begin{aligned} D(t) = \frac{2}{N(N-1)}\sum _{i=1}^{N-1}\sum _{j=i+1}^{N}\Vert x_i(t) - x_j(t)\Vert \end{aligned}$$

The diversity reduction follows an exponential decay pattern correlated with the inertia weight schedule:

102 $$\begin{aligned} D(t) \approx D_0 \cdot e^{-\alpha t} + D_{min} \end{aligned}$$

where $\alpha = 0.045$ is the decay rate and $D_{min} = 0.08$ is the minimum maintained diversity.

#### Convergence termination criteria

The optimization terminates when any of the following criteria is satisfied:

1. Maximum iterations reached: $t \ge T_{max} = 50$
2. Fitness stagnation: $|F(t) - F(t-5)| < \epsilon _F = 0.001$ for 5 consecutive iterations
3. Diversity collapse: $$D(t) < D_{threshold} = 0.05$$
4. Target fitness achieved: $F(t) \ge F_{target} = 0.95$

In 30 independent runs, termination occurred by: maximum iterations (63.3%), fitness stagnation (33.3%), and target achievement (3.4%). No runs terminated due to diversity collapse, confirming effective diversity preservation.

### Measurement uncertainty analysis

To ensure the validity and reproducibility of the experimental results, a comprehensive measurement uncertainty analysis was conducted following the guidelines established in the Guide to the Expression of Uncertainty in Measurement (GUM) . The uncertainty evaluation for all critical performance metrics, including voltage regulation, frequency stability, power measurement, and total harmonic distortion.

#### Instrumentation and measurement system

The experimental measurements were performed using calibrated instrumentation with traceable calibration certificates. Table 22 summarizes the specifications and accuracy classes of all measurement instruments employed in the experimental validation.

**Table 22:** Measurement Instrumentation Specifications.

Instrument	Model	Parameter	Accuracy
Power Analyzer	Tektronix PA4000	Voltage	$\pm 0.04\%$ rdg $+ 0.04\%$ rng
		Current	$\pm 0.04\%$ rdg $+ 0.04\%$ rng
		Power	$\pm 0.08\%$ rdg $+ 0.08\%$ rng
		Frequency	$\pm 0.01\%$ rdg
		THD	$\pm 0.1\%$ absolute
Voltage Sensor	LEM LV25-P	AC Voltage	$\pm 0.8\%$
Current Sensor	LEM LA55-P	AC Current	$\pm 0.65\%$
Oscilloscope	Keysight DSOX1204G	Voltage	$\pm 1.5\%$ vertical
		Time base	$\pm 0.005\%$
Temperature Sensor	PT100 RTD	Temperature	$\pm 0.5~^\circ$C
Speed Sensor	Incremental Encoder	Speed	$\pm 0.1\%$

#### Uncertainty evaluation methodology

The combined standard uncertainty was evaluated using Type A (statistical) and Type B (non-statistical) methods. For a measurement quantity *Y* dependent on input quantities $X_i$, the combined standard uncertainty is calculated as:

103 $$\begin{aligned} u_c(Y) = \sqrt{\sum _{i=1}^{n} \left( \frac{\partial Y}{\partial X_i} \right) ^2 u^2(X_i) + 2\sum _{i=1}^{n-1}\sum _{j=i+1}^{n} \frac{\partial Y}{\partial X_i} \frac{\partial Y}{\partial X_j} u(X_i, X_j)} \end{aligned}$$

where $u(X_i)$ is the standard uncertainty of input $X_i$, and $u(X_i, X_j)$ represents the covariance between correlated inputs.

For uncorrelated inputs, the simplified expression becomes:

104 $$\begin{aligned} u_c(Y) = \sqrt{\sum _{i=1}^{n} c_i^2 \cdot u^2(X_i)} \end{aligned}$$

where $c_i = \partial Y / \partial X_i$ are the sensitivity coefficients.

The expanded uncertainty *U* at a specified confidence level is obtained by multiplying the combined standard uncertainty by a coverage factor *k*:

105 $$\begin{aligned} U = k \cdot u_c(Y) \end{aligned}$$

For a 95% confidence level with sufficient degrees of freedom, $k = 2$ is applied throughout this analysis.

#### Type B uncertainty evaluation

Type B uncertainties were derived from instrument specifications, calibration certificates, and manufacturer data. Table 23 presents the Type B standard uncertainty for each measurement parameter.

**Table 23:** Type B Standard Uncertainty Evaluation.

Parameter	Nominal	Accuracy	Distribution	$u_B$
Voltage (V)	400 V	$\pm 0.04\%$ rdg	Rectangular	0.092 V
		$+ 0.04\%$ rng		
Sensor gain	—	$\pm 0.8\%$	Rectangular	1.85 V
Combined $u_B(V)$				1.86 V
Current (I)	4.8 A	$\pm 0.04\%$ rdg	Rectangular	0.0011 A
		$+ 0.04\%$ rng		
Sensor gain	—	$\pm 0.65\%$	Rectangular	0.018 A
Combined $u_B(I)$				0.018 A
Frequency (f)	50 Hz	$\pm 0.01\%$	Rectangular	0.0029 Hz
PLL uncertainty	—	$\pm 0.1\%$	Normal	0.025 Hz
Combined $u_B(f)$				0.025 Hz
Power (P)	2200 W	$\pm 0.08\%$ rdg	Rectangular	1.02 W
		$+ 0.08\%$ rng		
Combined $u_B(P)$				1.44 W
THD	5.8%	$\pm 0.1\%$ abs	Rectangular	0.058%
Combined $u_B(THD)$				0.058%

For rectangular distributions (uniform probability within bounds $\pm a$), the standard uncertainty is calculated as:

106 $$\begin{aligned} u_B = \frac{a}{\sqrt{3}} \end{aligned}$$

For normal distributions with specified confidence interval, the standard uncertainty is:

107 $$\begin{aligned} u_B = \frac{a}{k} \end{aligned}$$

where *k* corresponds to the coverage factor of the specified interval.

#### Type A uncertainty evaluation

Type A uncertainties were evaluated through repeated measurements under identical operating conditions. A series of $n = 10$ independent measurements was performed for each critical parameter at steady-state operation (full load, 50 Hz, 400 V reference).

The experimental mean value $\bar{x}$ and standard deviation *s* were calculated as:

108 $$\begin{aligned} \bar{x} = \frac{1}{n}\sum _{i=1}^{n} x_i, \quad s = \sqrt{\frac{1}{n-1}\sum _{i=1}^{n}(x_i - \bar{x})^2} \end{aligned}$$

The Type A standard uncertainty of the mean is:

109 $$\begin{aligned} u_A(\bar{x}) = \frac{s}{\sqrt{n}} \end{aligned}$$

Table 24 presents the repeatability analysis results for all critical parameters.

**Table 24:** Type A Uncertainty from Repeatability Analysis ($n = 10$ measurements).

Parameter	Mean $\bar{x}$	Std. Dev. *s*	$u_A$	Min.	Max.
Voltage (V)	399.8 V	1.82 V	0.58 V	396.9 V	402.4 V
Frequency (Hz)	49.97 Hz	0.031 Hz	0.010 Hz	49.91 Hz	50.02 Hz
Power (W)	2186 W	12.4 W	3.92 W	2168 W	2205 W
THD (%)	5.78%	0.14%	0.044%	5.58%	5.98%
Efficiency (%)	92.1%	0.42%	0.13%	91.4%	92.8%

#### Combined and expanded uncertainty

The combined standard uncertainty for each parameter was calculated by combining Type A and Type B contributions in quadrature:

110 $$\begin{aligned} u_c = \sqrt{u_A^2 + u_B^2} \end{aligned}$$

Table 25 presents the complete uncertainty budget for all experimental measurements.

**Table 25:** Complete Uncertainty Budget for Experimental Measurements.

Parameter	Measured value	$u_A$	$u_B$	$u_c$	*U* ($k=2$)	Relative uncertainty	DOF $\nu _{eff}$
Terminal Voltage	399.8 V	0.58 V	1.86 V	1.95 V	3.9 V	0.98%	28
Frequency	49.97 Hz	0.010 Hz	0.025 Hz	0.027 Hz	0.054 Hz	0.11%	19
Output Power	2186 W	3.92 W	1.44 W	4.18 W	8.4 W	0.38%	42
Voltage THD	5.78%	0.044%	0.058%	0.073%	0.15%	2.5% (rel.)	35
Current THD	3.82%	0.038%	0.052%	0.064%	0.13%	3.4% (rel.)	31
DC-link Voltage	586.2 V	0.72 V	2.34 V	2.45 V	4.9 V	0.84%	24
Energy Recovery Eff.	92.1%	0.13%	0.35%	0.37%	0.74%	0.80%	22

DOF: Effective degrees of freedom calculated using Welch–Satterthwaite formula

The effective degrees of freedom $\nu _{eff}$ were calculated using the Welch-Satterthwaite formula:

111 $$\begin{aligned} \nu _{eff} = \frac{u_c^4}{\sum _{i=1}^{n} \frac{u_i^4}{\nu _i}} \end{aligned}$$

where $\nu _i$ is the degrees of freedom associated with each uncertainty component ($\nu = n-1$ for Type A, $\nu = \infty$ for Type B from specifications).

#### Uncertainty in derived quantities

For performance metrics derived from multiple measured quantities, uncertainty propagation was applied. The voltage regulation accuracy is calculated as:

112 $$\begin{aligned} \epsilon _V = \frac{|V_{ref} - V_{meas}|}{V_{ref}} \times 100\% \end{aligned}$$

The uncertainty in voltage regulation is:

113 $$\begin{aligned} u(\epsilon _V) = \frac{1}{V_{ref}} \sqrt{u^2(V_{meas}) + \left( \frac{V_{meas}}{V_{ref}}\right) ^2 u^2(V_{ref})} \end{aligned}$$

Assuming $u(V_{ref}) \approx 0$ (reference is exact), the uncertainty simplifies to:

114 $$\begin{aligned} u(\epsilon _V) = \frac{u(V_{meas})}{V_{ref}} \times 100\% = \frac{1.95}{400} \times 100\% = 0.49\% \end{aligned}$$

Thus, the reported voltage regulation accuracy of $\pm 1.8\%$ has an expanded uncertainty of $U(\epsilon _V) = \pm 0.98\%$, yielding the final result:

115 $$\begin{aligned} \epsilon _V = (1.8 \pm 1.0)\%, \quad k = 2, \quad 95\% \text { confidence} \end{aligned}$$

Similarly, for frequency stability:

116 $$\begin{aligned} \epsilon _f = (0.9 \pm 0.11)\%, \quad k = 2, \quad 95\% \text { confidence} \end{aligned}$$

#### Comparison with performance improvements

To validate the statistical significance of the reported performance improvements, the measurement uncertainties were compared with the claimed improvements over conventional methods. Table 26 presents this comparison.

**Table 26:** Statistical Significance of Performance Improvements.

Metric	Conv.	PSO	Improvement	Significant?
	Method	(Proposed)		($U < \Delta$)
Voltage reg. (%)	$\pm 8.0$	$\pm 1.8 \pm 1.0$	6.2%	**Yes**
Frequency stab. (%)	$\pm 3.0$	$\pm 0.9 \pm 0.11$	2.1%	**Yes**
Voltage THD (%)	10.1	$5.78 \pm 0.15$	4.32%	**Yes**
Current THD (%)	15.6	$3.82 \pm 0.13$	11.78%	**Yes**
Settling time (s)	1.20	$0.31 \pm 0.04$	0.89 s	**Yes**
Energy recovery (%)	0	$92.1 \pm 0.74$	92.1%	**Yes**

The analysis confirms that all reported performance improvements are statistically significant, with the improvement magnitude exceeding the expanded measurement uncertainty by factors ranging from 4.2 (voltage regulation) to 124.5 (energy recovery). This validates the claimed superiority of the proposed PSO-based ELC over conventional methods.

## Conclusion

This paper presented a novel multi-objective Particle Swarm Optimization based Electronic Load Controller with intelligent energy recovery for standalone Self-Excited Induction Generator based micro-hydro power systems. The research addresses critical challenges in rural electrification by simultaneously optimizing voltage regulation, frequency stability, harmonic distortion, and energy recovery through productive dump load utilization.

The proposed approach introduces three significant scientific contributions that advance the current state-of-the-art in SEIG-based power generation. The first contribution is the development of a multi-objective optimization algorithm that simultaneously addresses four competing objectives through an adaptive weight mechanism derived using the Analytical Hierarchy Process. Unlike conventional single-objective approaches that optimize voltage regulation in isolation while neglecting other performance aspects, the proposed method achieves balanced trade-offs between power quality and energy efficiency within a unified optimization structure. The PSO algorithm employs time-varying cognitive and social coefficients with nonlinear inertia weight decay, enabling effective exploration of the ten-dimensional search space comprising voltage controller gains, frequency controller gains, PWM parameters, and pump power allocation. The second contribution is the paradigm shift from energy dissipation to productive energy utilization through integration of water pumping as a controllable dump load. Conventional ELC designs employ resistive dump loads that waste up to 40% of generated energy as heat, representing a fundamental inefficiency that this work overcomes by channeling excess power to productive applications. The water pumping system operates as a variable load whose power consumption is optimized in real-time based on excess generation availability, system constraints, and pump efficiency characteristics. The third contribution is the demonstration of real-time implementation feasibility on practical embedded controllers. The proposed PSO algorithm achieves convergence within 15.2 iterations with an average execution time of 0.83 milliseconds per optimization cycle, which is well within the 10 millisecond control period required for effective voltage and frequency regulation. This computational efficiency enables deployment on standard industrial controllers without specialized hardware, bridging the gap between academic optimization research and practical field implementation. The experimental validation on a 2.2 kW laboratory prototype demonstrates significant performance improvements across all evaluated metrics. The proposed PSO-optimized ELC achieves voltage regulation accuracy of ±1.8% compared to ±8.0% for conventional PI-based controllers, representing an improvement of 77.5%. The frequency stability of ±0.9% shows 70.0% improvement over the conventional method’s ±3.0% deviation. The voltage and current total harmonic distortion values of 4.2% and 3.8% respectively comply with IEEE 519 power quality standards, demonstrating reductions of 58.4% and 75.6% compared to conventional approaches. The comparative analysis with other metaheuristic algorithms confirms the superiority of PSO, achieving 46.7% faster convergence than Genetic Algorithm, 31.8% faster than Grey Wolf Optimizer, 52.1% faster than Differential Evolution, and 41.1% faster than Whale Optimization Algorithm. The energy recovery efficiency of 92.1% through water pumping translates to approximately 3.2 million liters of water pumped annually for a typical operating profile, generating estimated cost savings of $1,567 per year with a payback period of 2.1 years and annual carbon dioxide emission reduction of 5.2 tons. The system maintains robust stability with gain margin of 8.2 dB and phase margin of 52 degrees, while Monte Carlo simulation with 1000 trials demonstrates 100% stability success rate under ±15% parameter variations, confirming the robustness of the optimized controller parameters.

The research contributions carry significant implications for both the scientific community and practical applications in rural electrification. From a technical perspective, the multi-objective optimization methodology establishes a benchmark for SEIG control system design and provides a replicable approach for other distributed generation systems including wind turbines, small-scale hydropower, and hybrid renewable energy installations. The comprehensive stability analysis incorporating eigenvalue assessment, Lyapunov stability proof, and robust stability evaluation using structured singular value analysis offers rigorous analytical support that can be extended to other nonlinear power electronic systems. The sensitivity analysis methodology for both PSO parameters and control system parameters provides guidelines for practitioners implementing similar optimization-based controllers. From a societal perspective, the proposed system directly addresses United Nations Sustainable Development Goals related to affordable and clean energy access and clean water availability. The simultaneous provision of electricity and pumped water through a single generation system significantly enhances the value proposition for micro-hydro installations in remote communities where both resources are scarce. The elimination of grid dependency enables electrification of isolated areas where transmission infrastructure is economically infeasible, potentially benefiting the estimated 675 million people worldwide who currently lack electricity access. From an economic perspective, the productive utilization of excess energy fundamentally improves project viability by generating additional revenue streams or cost savings that offset installation costs. The demonstrated payback period of 2.1 years makes small-scale micro-hydro installations attractive to investors, community cooperatives, and development organizations seeking sustainable rural infrastructure solutions. Several limitations of the current study are acknowledged to provide context for the reported findings and guide future research. The experimental validation was performed on a single 2.2 kW laboratory prototype, and performance scaling to higher power ratings in the range of 10 to 100 kW requires further investigation to confirm that the optimization approach and controller parameters translate effectively to larger systems. The pump load characteristics analyzed in this study were specific to centrifugal pumps with quadratic torque-speed relationships, and other productive load types such as positive displacement pumps, battery charging systems, or heating elements may require modified control strategies and constraint formulations. The experimental conditions represented controlled laboratory environment with stable prime mover speed and predictable load variations, whereas field deployment would encounter additional challenges including varying water head, sediment-induced turbine wear, ambient temperature fluctuations, and unpredictable consumer load patterns. The communication and data logging infrastructure required for the OPAL-RT based implementation may not be readily available in remote rural locations, necessitating development of standalone embedded controller solutions for practical deployment. Long-term reliability assessment spanning multiple years of continuous operation was not conducted, and component degradation effects on control performance remain to be evaluated.

Future research emerging from this work can be structured across short-, medium-, and long-term horizons to ensure both technical advancement and practical impact. In the short term (1–2 years), emphasis should be placed on field deployment and validation of the proposed system in real micro-hydro installations to assess performance under variable hydrological conditions, load dynamics, and environmental influences, along with scaling studies from the current 2.2 kW prototype to community-scale capacities of 10–50 kW. Integration of battery energy storage systems should be explored to enhance power quality during transients and provide energy buffering, requiring extension of the optimization framework to include state-of-charge management, while parallel efforts should focus on developing low-cost embedded controller implementations using DSPs or FPGAs for practical rural deployment. In the medium term (2–5 years), research should address coordinated operation of multiple parallel SEIG units in micro-grid configurations through distributed optimization strategies, complemented by hybrid PSO–machine learning approaches for adaptive control, load forecasting, predictive pump scheduling, and condition-based maintenance. Investigation of alternative productive loads, such as agro-processing, cold-chain applications, and other rural industries, would further extend the applicability of the proposed energy recovery concept. In the long term (beyond 5 years), efforts should focus on standardization and commercialization, smart-grid integration with remote monitoring and demand response capabilities, and fully autonomous operation using artificial intelligence for self-tuning and fault management, supported by comprehensive techno-economic, lifecycle, and policy analyses to guide large-scale adoption of micro-hydro electrification solutions.

## Supplementary Information

Supplementary Information.

## Data Availability

The data that support the findings of this study are openly available in “figshare” at https://doi.org/10.6084/m9.figshare.29712599.v1, reference number 29712599^38^.

## Parameters of SEIG and 1-phase induction motor load

**Parameters of SEIG (2.2 kW)** A 415 V, 4.8 A, 50 Hz, 4-pole, delta-connected three-phase squirrel-cage induction machine is used as the SEIG. The electrical and mechanical parameters obtained through no-load and blocked rotor tests are listed below:

- $$R_s = 3.81~\Omega$$,    $$R_r = 2.87~\Omega$$
- $$L_s = L_r = 0.014196~\text {H}$$,    $$L_m = 0.3012707~\text {H}$$
- $$X_s = X_r = 4.457544~\Omega$$,    $$X_m = 94.5989~\Omega$$
- Moment of inertia, $J = 0.00759~\text {kg}\cdot \text {m}^2$

The machine parameters yield a saturation curve. The magnetization characteristics of the 2.2 kW machine are expressed by the following equations:

$$\begin{aligned} \frac{V_{ag}}{F_{pu}}&= -43.252 + 258.61 I_{mg} - I_{mg}^2 \\ L_{mg}&= 0.330 + 0.016 I_{mg} - 0.014 I_{mg}^2 \end{aligned}$$

**Parameters of the 1-phase induction motor load** The single-phase induction motor is a *Self-Priming Regenerative Pump* with the following specifications:

- Pump No.: LPEC1WH031906
- Rated Speed: 2780 RPM
- Voltage: 220 V,    Current: 2.50 A
- Power: 0.37 kW (0.50 HP)
- Head Range: 6–21 m
