# Supplementary material for: PSO-optimized electronic load controller with intelligent energy recovery for self-excited induction generator based micro-hydro systems
Source: Sci Rep. 2026 Mar 27;16:10862. doi: 10.1038/s41598-026-45570-6 (PMC13039360; doi:10.1038/s41598-026-45570-6)
Supplement: Supplementary file 1 — Supplementary Information. [file 41598_2026_45570_MOESM1_ESM.pdf]

Supplementary Material for: PSO-Optimized Electronic Load Controller with Intelligent Energy Recovery for Self-Excited Induction Generator Based Micro-Hydro Systems

Shalini Sinha[1], Mrinal Kanti Rajak[1], Rajen Pudur[1]
[1]Department of Electrical Engineering,
National Institute of Technology Arunachal Pradesh, India
Email: mrinal.phd20@nitap.ac.in

1 PSO Algorithm Data

Table 1. PSO Algorithm Configuration Details

**Parameter**	**Symbol**	**Initial Value / Final Value**	**Update Rule / Remarks**
Population Size	N	20 / 20	$N = 10 + 2\sqrt{D}$, where $D = 10$
Inertia Weight	$w(t)$	0.9 / 0.4	$w(t) = w_{\max} - (w_{\max} - w_{\min})(t/t_{\max})^{\alpha}$
Inertia Alpha	α	1.5 / 1.5	Nonlinear decrement factor
Cognitive Coefficient	$c_1(t)$	2.5 / 0.5	$c_1(t) = c_{1,\max} - (c_{1,\max} - c_{1,\min})(t/t_{\max})$
Social Coefficient	$c_2(t)$	0.5 / 2.5	$c_2(t) = c_{2,\min} + (c_{2,\max} - c_{2,\min})(t/t_{\max})$
Velocity Clamping	$V_{\max}$	±20% of range	Per-dimension velocity limits
Boundary Handling	–	Reflection	$X_{\text{new}} = 2 \times \text{boundary} - X_{\text{old}}$
Diversity Threshold	$D_{\min}$	0.01 / 0.01	Early termination criterion

Table 2. Multi-Objective Fitness Function Weights

Weight	Symbol	Base Value / Adaptive Range	Update Formula / Remarks
Voltage Weight	$w_1(t)$	0.25 / 0.15–0.40	$w_{1,\text{base}}(1+\kappa_1)$
Frequency Weight	$w_2(t)$	0.25 / 0.15–0.40	$w_{2,\text{base}}(1+\kappa_2)$
THD Weight	$w_3(t)$	0.25 / 0.15–0.40	$w_{3,\text{base}}(1+\kappa_3 \cdot \frac{\text{THD}}{\text{THD}_{\text{limit}}})$
Energy Weight	$w_4(t)$	0.25 / 0.10–0.35	$w_{4,\text{base}} \cdot \left(\frac{P_{\text{surplus}}}{P_{\text{rated}}}\right)$
Adaptation Factor	κ_1, κ_2, κ_3	0.5 / 0.1–1.0	Real-time error sensitivity
Weight Smoothing	α_i	0.8 / 0.7–0.9	$w_i(t+1) = \alpha_i \cdot w_i(t) + (1-\alpha_i) \cdot w_{i,\text{desired}}$

Table 3. PSO Search Space Boundaries

Variable	Symbol	Lower Bound / Upper Bound	Physical Constraint / Remarks
Voltage Proportional Gain	$K_{p,v}$	0.1 / 10.0	Stability margin
Voltage Integral Gain	$K_{i,v}$	0.01 / 5.0	Steady-state error
Voltage Derivative Gain	$K_{d,v}$	0.0 / 2.0	Noise sensitivity
Frequency Proportional Gain	$K_{p,f}$	0.1 / 8.0	Dynamic response
Frequency Integral Gain	$K_{i,f}$	0.01 / 4.0	Frequency droop
Frequency Derivative Gain	$K_{d,f}$	0.0 / 1.5	Derivative kick
Modulation Index	m_a	0.1 / 0.95	VSI linearity
Phase Angle	θ_{phase}	$-\pi/6$ / $\pi/6$	Power factor
Switching Frequency	f_c	1000 / 50000	Hz, switching losses
Pump Reference Power	$P_{\text{pump,ref}}$	0 / $P_{\text{gen}} - P_{\text{load}} - P_{\text{loss}}$	Available power

Table 4. PSO Convergence Statistics

Iteration	Best Fitness	Average Fitness	Worst Fitness	Diversity (%)	Convergence Rate
10	0.537	0.402	0.185	100.0	–

Table 14 (continued): PSO Convergence Statistics

Iteration	Best Fitness	Average Fitness	Worst Fitness	Diversity (%)	Convergence Rate
50	0.672	0.548	0.321	78.4	0.135
100	0.758	0.681	0.452	45.2	0.086
150	0.834	0.786	0.598	28.7	0.076
200	0.871	0.842	0.691	18.3	0.037
250	0.888	0.867	0.732	12.6	0.017
300	0.896	0.881	0.758	9.4	0.008
400	0.901	0.894	0.784	6.8	0.005
500	0.903	0.898	0.796	4.2	0.002

Table 5. Individual Objective Function Convergence

Objective	Initial	Final	Improvement (%)	Time Constant	Convergence Model
Voltage Fitness	0.30	0.92	206.7%	6.67 iter	$0.3 + 0.6e^{-0.15t}$
Frequency Fitness	0.25	0.92	268.0%	8.33 iter	$0.25 + 0.65e^{-0.12t}$
THD Fitness	0.20	0.93	365.0%	5.56 iter	$0.2 + 0.7e^{-0.18t}$
Energy Fitness	0.10	0.98	880.0%	10.0 iter	$0.1 + 0.8e^{-0.10t}$

Table 6. PSO Velocity Update Parameters

Particle	Velocity	Initial Value	Maximum Value	Update Frequency	Clamping Method
All	$V_{K_{p,v}}$	±2.0	±4.0	100 Hz	Symmetric clamping
All	$V_{K_{i,v}}$	±1.0	±2.0	100 Hz	Symmetric clamping
All	$V_{K_{d,v}}$	±0.4	±0.8	100 Hz	Symmetric clamping
All	$V_{K_{p,f}}$	±1.6	±3.2	100 Hz	Symmetric clamping
All	$V_{K_{i,f}}$	±0.8	±1.6	100 Hz	Symmetric clamping
All	$V_{K_{d,f}}$	±0.3	±0.6	100 Hz	Symmetric clamping
All	V_{m_a}	±0.17	±0.34	100 Hz	Symmetric clamping
All	$V_{\theta_{\text{phase}}}$	±0.52	±1.05	100 Hz	Symmetric clamping
All	V_{f_c}	±9800	±19600	100 Hz	Symmetric clamping
All	$V_{P_{\text{pump}}}$	±440	±880	100 Hz	Symmetric clamping

Table 7. PSO Performance Metrics by Iteration

Metric	Iter 1–10	Iter 11–20	Iter 21–30	Iter 31–40	Iter 41–50
Exploration Rate (%)	85%	65%	45%	25%	15%

Table 17 (continued): PSO Performance Metrics by Iteration

**Metric**	**Iter 1–10**	**Iter 11–20**	**Iter 21–30**	**Iter 31–40**	**Iter 41–50**
Exploitation Rate (%)	15%	35%	55%	75%	85%
Stagnation Count	0	1	3	5	8
Best Update Frequency (%)	90%	70%	40%	20%	10%
Diversity Loss Rate (%/iter)	8%/iter	6%/iter	4%/iter	2%/iter	1%/iter
CPU Usage (%)	72%	68%	65%	63%	61%

Table 8. Adaptive PSO Parameters

**Parameter**	**Adaptive Formula**	**Initial**	**Mid-point**	**Final**	**Purpose**
Inertia Weight $w(t)$	$w(t) = w_{\max} - (w_{\max} - w_{\min}) \left(\frac{t}{t_{\max}}\right)^{1.5}$	0.9	0.65	0.4	Balance exploration
Cognitive Factor $c_1(t)$	$c_1(t) = 2.5 \left(1 + e^{\gamma \frac{(t - t_{\text{mid}})}{t_{\max}}}\right)^{-1} + 0.5$	2.5	1.5	0.5	Reduce self-confidence
Social Factor $c_2(t)$	$c_2(t) = 0.5 + 2.0 \left(1 - e^{-\delta t / t_{\max}}\right)$	0.5	1.5	2.5	Increase swarm influence
Diversity Monitor $D(t)$	$D(t) = \frac{1}{ND} \sum_{i=1}^{N} \sum_{j=1}^{D} (X_{i,j} - \bar{X}_j)^2$	100%	35%	8%	Population spread
Convergence Rate $CR(t)$	$CR(t) = \frac{F(t) - F(t-5)}{F(t-5)}$	0.15	—	0.05	Swarm improvement tracking

Table 9. PSO Memory and Computational Requirements

**Component**	**Memory Usage**	**CPU Cycles**	**Update Frequency**	**Data Type**
Particle Positions	800 bytes	200/particle	100 Hz	`float32[20×10]`
Particle Velocities	800 bytes	200/particle	100 Hz	`float32[20×10]`
Personal Best	800 bytes	50/particle	Variable	`float32[20×10]`
Global Best	40 bytes	20	Variable	`float32[10]`
Fitness Values	80 bytes	1000/particle	100 Hz	`float32[20]`
Constraint Penalties	80 bytes	100/particle	100 Hz	`float32[20]`
Total Memory	2.6 kB	—	—	—
Total CPU Load	—	67% avg	—	—

Table 10. PSO Stopping Criteria

**Criterion**	**Threshold**	**Mathematical Expression**	**Trigger Frequency**
Fitness Convergence	0.001	$\lvert F(\text{Gbest}, t) - F(\text{Gbest}, t{-}1) \rvert < \varepsilon_{\text{conv}}$	95% of cases
Maximum Iterations	50	$t \geq t_{\max}$	3% of cases
Diversity Loss	0.01	$D(t) < D_{\min}$	2% of cases
Stagnation Limit	15	No improvement for 15 iterations	¡1% of cases
Time Limit	50 ms	Real-time constraint	Emergency only

Table 11. PSO Particle Position Examples (Selected Iterations)

**Particle**	**Iter**	K_{pv}	K_{iv}	K_{dv}	K_{pf}	K_{if}	K_{df}	m_a	θ_{phase}	f_c	P_{pump}	**Fitness**
1	12	2.3	1.2	0.5	3.1	0.8	0.3	0.75	0.11	15000	8000	0.542
1	25	4.2	2.1	0.8	4.5	1.5	0.6	0.82	0.05	18500	12000	0.887
1	50	5.1	2.8	1.1	5.2	2.1	0.9	0.85	0.02	20000	15000	0.903
**Best**	50	5.1	2.8	1.1	5.2	2.1	0.9	0.85	0.02	20000	15000	0.903

Table 12. PSO Algorithm Validation Metrics

**Test Case**	**Load Condition**	**Convergence Time**	**Final Fitness**	**Voltage Error**	**Frequency Error**
Test 1	25% Load	12.8 iter	0.921	±1.6%	±0.7%
Test 2	50% Load	14.5 iter	0.903	±1.8%	±0.9%
Test 3	75% Load	16.2 iter	0.895	±2.1%	±1.1%
Test 4	100% Load	18.1 iter	0.887	±2.3%	±1.3%
Test 5	Variable Load	15.2 iter	0.903	±1.8%	±0.9%
Test 6	Transient Load	13.7 iter	0.915	±1.5%	±0.8%

Table 13. Real-Time PSO Implementation Details

**Aspect**	**Specification**	**Value / Implementation Notes**
Sampling Frequency	Control Loop	10 kHz (PWM generation)
Optimization Frequency	PSO Loop	100 Hz (Parameter updates)
Communication Buffer	Size	512 bytes (Inter-loop data)
Synchronization Method	Type	Buffered (Seamless updates)
Memory Management	Type	Static allocation (Real-time safety)
Floating Point	Precision	32-bit (Computational accuracy)
Execution Time	PSO Iteration	0.83 ms (Measured average)
Worst Case Execution Time	-	1.2 ms (Maximum observed)
